# Combining Comparative Proteomics and Molecular Genetics Uncovers Regulators of Synaptic and Axonal Stability and Degeneration *In Vivo*

**DOI:** 10.1371/journal.pgen.1002936

**Published:** 2012-08-30

**Authors:** Thomas M. Wishart, Timothy M. Rooney, Douglas J. Lamont, Ann K. Wright, A. Jennifer Morton, Mandy Jackson, Marc R. Freeman, Thomas H. Gillingwater

**Affiliations:** 1 Centre for Integrative Physiology, University of Edinburgh, Edinburgh, United Kingdom; 2 Euan MacDonald Centre for Motor Neurone Disease Research, University of Edinburgh, Edinburgh, United Kingdom; 3 Division of Neurobiology, The Roslin Institute and Royal (Dick) School of Veterinary Studies, University of Edinburgh, Edinburgh, United Kingdom; 4 Department of Neurobiology, Howard Hughes Medical Institute, University of Massachusetts Medical School, Worcester, Massachusetts, United States of America; 5 FingerPrints Proteomics Facility, College of Life Sciences, University of Dundee, Dundee, United Kingdom; 6 Department of Pharmacology, University of Cambridge, Cambridge, United Kingdom; University of California San Diego, United States of America

## Abstract

Degeneration of synaptic and axonal compartments of neurons is an early event contributing to the pathogenesis of many neurodegenerative diseases, but the underlying molecular mechanisms remain unclear. Here, we demonstrate the effectiveness of a novel “top-down” approach for identifying proteins and functional pathways regulating neurodegeneration in distal compartments of neurons. A series of comparative quantitative proteomic screens on synapse-enriched fractions isolated from the mouse brain following injury identified dynamic perturbations occurring within the proteome during both initiation and onset phases of degeneration. *In silico* analyses highlighted significant clustering of proteins contributing to functional pathways regulating synaptic transmission and neurite development. Molecular markers of degeneration were conserved in injury and disease, with comparable responses observed in synapse-enriched fractions isolated from mouse models of Huntington's disease (HD) and spinocerebellar ataxia type 5. An initial screen targeting thirteen degeneration-associated proteins using mutant *Drosophila* lines revealed six potential regulators of synaptic and axonal degeneration *in vivo*. Mutations in CALB2, ROCK2, DNAJC5/CSP, and HIBCH partially delayed injury-induced neurodegeneration. Conversely, mutations in DNAJC6 and ALDHA1 led to spontaneous degeneration of distal axons and synapses. A more detailed genetic analysis of DNAJC5/CSP mutants confirmed that loss of DNAJC5/CSP was neuroprotective, robustly delaying degeneration in axonal and synaptic compartments. Our study has identified conserved molecular responses occurring within synapse-enriched fractions of the mouse brain during the early stages of neurodegeneration, focused on functional networks modulating synaptic transmission and incorporating molecular chaperones, cytoskeletal modifiers, and calcium-binding proteins. We propose that the proteins and functional pathways identified in the current study represent attractive targets for developing therapeutics aimed at modulating synaptic and axonal stability and neurodegeneration *in vivo*.

## Author Summary

In diseases affecting the nervous system, such as Alzheimer's disease and motor neuron disease, the breakdown of synaptic connections between neurons is a critical early event, contributing to disease onset and progression. However, we still know very little about the molecular machinery present in synaptic and axonal compartments of neurons that regulate their stability and cause breakdown during neurodegeneration. In this study we examined the protein composition of healthy and degenerating synapse-enriched fractions isolated from the brains of mice in order to identify early molecular changes occurring during neurodegeneration. We identified a range of proteins and cellular pathways that were modulated in synapse-enriched fractions during the early phases of degeneration, many of which were already known to regulate synaptic function. Similar molecular alterations were found in synapse-enriched fractions prepared from mouse models of Huntington's disease (HD) and spinocerebellar ataxia type 5. Data from these proteomic studies were then used to design experiments in *Drosophila*, in which we found that at least six of the individual proteins modified in degenerating synapses from mice were capable of independently regulating neuronal stability and degeneration *in vivo*. Designing novel therapeutics to target these proteins and pathways may help to delay or prevent neurodegeneration across a range of diseases.

## Introduction

Synaptic and axonal compartments of neurons are exceptionally vulnerable to a wide array of neurodegenerative stimuli, ranging from physical trauma through to genetic disease [1]. As a result, the important role that synapses and axons play in the initiation and progression of a wide range of neurodegenerative conditions is becoming increasingly well documented. For example, published studies have highlighted an important role for synaptic malfunction and degeneration in pre-clinical and early-symptomatic stages of Alzheimer's disease [2], Parkinson's disease [3], Huntington's disease (HD) [4], spinocerebellar ataxia [5], prion diseases [6], lysosomal storage disorders [7] and motor neuron diseases [8], [9]. In many such conditions, synaptic and axonal pathology is instigated in advance of pathological changes in other regions of the neuron (e.g. the cell soma). Thus, neuroprotective strategies directly targeting synapses and axons are likely to provide important options for treating neurodegenerative disorders in human patients [1], [10], [11].

Despite an increasing awareness of the scientific and clinical importance of synaptic and axonal degeneration, little is known about why distal compartments of neurons are particularly vulnerable. Furthermore, our understanding of molecular and genetic mechanisms regulating neurodegeneration remains in its infancy. Of the many proteins present in synapses and distal axons, only a few have been shown to be capable of directly modulating neurodegeneration. One of the most extensively characterised, the chimeric Wallerian degeneration slow (*Wld^S^*) protein [12]–[14], is encoded by a novel chimeric gene formed by a spontaneous mutation event in laboratory mice. It is not, therefore, endogenously expressed in other species, including humans. Examples of endogenous proteins capable of modulating synapse and distal axon degeneration *in vivo* are relatively rare, including cysteine string protein alpha (also known as DNAJC5) [15] and some synucleins [16], [17] (for review see [11]). There is, therefore, a need to identify other proteins and pathways capable of modulating synaptic and axonal stability and degeneration *in vivo*. However, this is likely to require the development of integrated experimental approaches capable of identifying and characterizing molecular responses to degeneration in distal compartments of neurons.

Here, we report on the development of a novel ‘top-down’ approach for identifying proteins and functional pathways regulating neurodegeneration in distal compartments of neurons *in vivo*. We combined sequential comparative proteomic screens on synapse-enriched fractions isolated from the mouse brain undergoing injury-induced degeneration with molecular genetic dissection of mechanisms underlying degeneration in *Drosophila*. We show that synaptic and axonal degeneration is associated with dynamic perturbations to the proteome, impacting on molecular pathways involved with synaptic transmission and neurite development. Experiments on two mouse models of neurodegenerative disease (Huntington's disease and spinocerebellar ataxia type 5) showed that molecular pathways underlying distal neuron degeneration were conserved from injury to disease. Genetic manipulation of 13 synaptic proteins using mutant *Drosophila* lines led to the identification of 6 potential regulators of axonal and synaptic degeneration *in vivo*: ALDHA1 (Aldehyde dehydrogenase), CALB2 (calbindin2), DNAJC5/CSP (DnaJ (Hsp40) homolog, subfamily C, member 5), DNAJC6 (DnaJ (Hsp40) homolog, subfamily B, member 6), HIBCH (3-hydroxyisobutyryl-CoA hydrolase) and ROCK2 (Rho-associated, coiled-coil containing protein kinase 2). A more robust genetic analysis of DNAJC5/CSP confirmed that loss of this synaptic protein was neuroprotective, robustly delaying degeneration in axonal and synaptic compartments of neurons *in vivo*. We conclude that conserved molecular responses are instigated locally within distal compartments of neurons during the early stages of neurodegeneration. Such responses are focused around networks of proteins modulating synaptic transmission, incorporating molecular chaperones, cytoskeletal modifiers, and calcium binding proteins.

## Results

### Proteomic identification of molecular responses to degeneration in synapse-enriched fractions of the mouse brain

**Figure 1:**
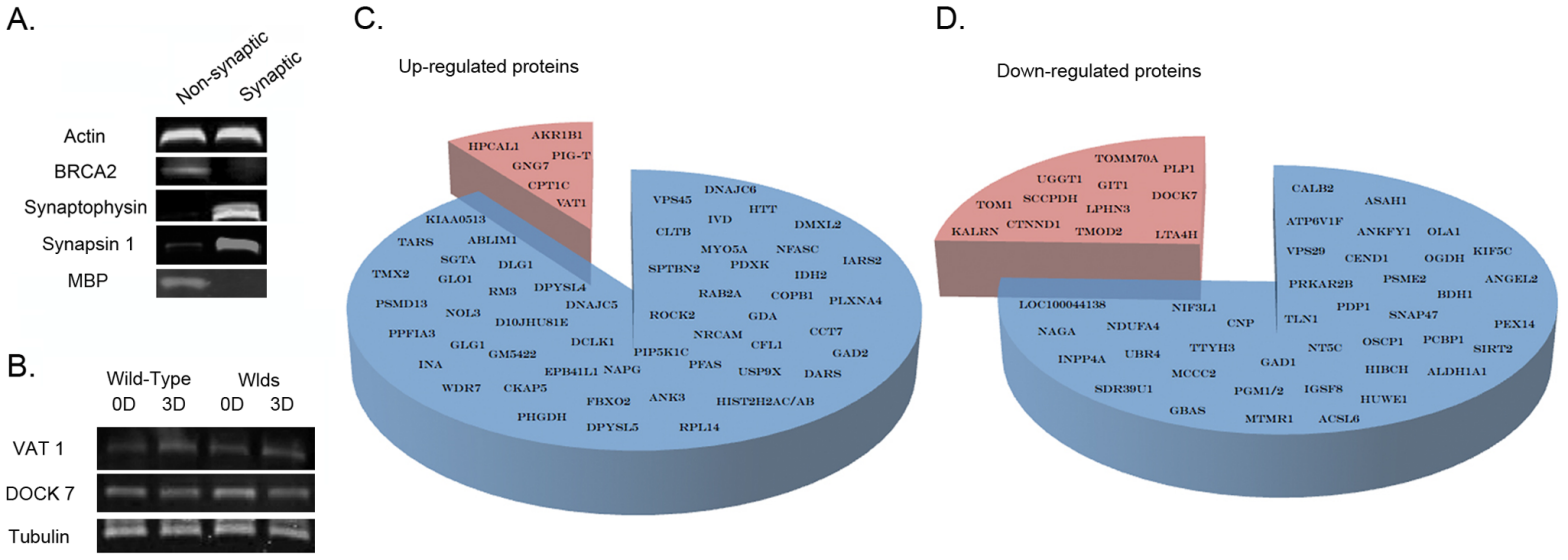
Proteomic identification and refinement of molecular pathways underlying degeneration in synapse-enriched brain fractions. A. Representative bands from fluorescent western blots demonstrating enrichment of two distinct synaptic proteins (synaptophysin and synapsin-1) and relative purity of the synapse-enriched fractions, comparatively free from nuclear contamination (BRCA2) and glial cell contamination (MBP) relative to non-synaptic fractions. Actin is shown as a loading control. B. Representative bands from fluorescent western blots showing examples of protein expression changes (VAT1 and DOCK7) present in striatal synapse-enriched fractions from both wild-type and *Wld^S^* mice before (0D) and 3 days after (3D) cortical lesion. Both proteins showed similar alterations in expression in degenerating tissue across wild-type and *Wld^S^* mice suggesting that they are more likely to represent systemic responses to injury rather than direct mediators of the degenerative process (as synaptic degeneration is yet to be initiated in *Wld^S^* mice 3 days after lesion [14]). Tubulin is shown as a loading control. C/D. Pie charts representing all proteins found to have altered expression >20% following cortical lesion. The blue section of each chart represents those proteins altered only in wild-type mice. The red sections show those proteins found to be altered in both wild-type and *Wld^S^* mice, which were subsequently subtracted from the proteomic profile as they were not considered to represent expression changes underlying degeneration.

To uncover molecular pathways activated in synapses and axons during the early stages of neurodegeneration we initially wanted to obtain a global overview of protein expression changes occurring locally within distal neuronal compartments undergoing degeneration in response to a defined stimulus. We therefore performed a series of comparative, unbiased proteomic screens on synapse-enriched fractions biochemically isolated from the mouse brain (see Methods and Figure 1A) [18]. The relative absence of nuclear proteins (BRCA2) and glial cell proteins (MBP), alongside robust levels of synaptic proteins synaptophysin and synapsin 1 (Figure 1A), confirmed the enrichment of synaptic material in these preparations. However, it should be noted that low-level contamination originating from other cell types and/or non-synaptic fractions is likely to be present in these preparations.

Synaptic and distal axon degeneration was induced using an *in vivo* cortical lesion model that injures cell bodies and proximal axons giving rise to corticostriatal projections. In this model unilateral ablation of one cortical hemisphere down to the level of the corpus callosum reliably triggers axonal and synaptic degeneration in the underlying ipsilateral striatum [14], [19]. Morphological evidence for axonal and synaptic degeneration is not observed until 48 hours following injury in wild-type mice (C57Bl/6) [14], [19], revealing the presence of a ∼24–48 hour lag period preceding the physical onset of degeneration. We therefore generated synapse-enriched fractions preparations from the striatum of wild-type mice at 3 time points (N = 6 mice per time point): prior to injury (0 hrs), providing a base-line for protein detection and expression; 24 hours following injury (24 hrs) to identify immediate early responses triggered during the initiation of degeneration; and 48 hours following injury (48 hrs), correlating with the onset of synaptic breakdown. In order to increase stringency in reporting, a minimum cut-off threshold of 20% change versus uninjured controls was used to indicate a protein with modified expression levels. iTRAQ (Isobaric Tag for Relative and Absolute Quantitation) proteomic analyses identified a total of 178 putative proteins (from a pool of 56,957 peptide sequences) with expression levels modified by more than 20% at either 24 h or 48 hr after injury compared to uninjured (0 hrs) controls. Of these, 112 putative proteins showed expression changes of greater than 20% maintained at 48 hrs following injury (Table S1). Bioinformatics analysis of the 178 putative proteins using IPA software revealed that >87% of the proteins were cytoplasmic or membrane bound, consistent with them being synaptic proteins, rather than arising as a result of contamination from nuclear compartments.

Modifications to the proteome of synapse-enriched fractions revealed in our initial analyses are unlikely to solely represent responses directly associated with degeneration, as the cortical lesion injury used generated systemic responses in corticostriatal networks that could conceivably effect the molecular composition of the tissue (e.g. in response to modified patterns of activity). In order to improve our dataset, leaving only molecular responses directly associated with active processes of degeneration, we refined our analysis by undertaking a subsequent comparison with proteomic data obtained from synapse-enriched fractions in mice genetically protected from neurodegeneration by the *Wld^s^* gene. We have previously demonstrated that both axonal and synaptic degeneration in the striatum are absent for at least 6 days after a cortical lesion in *Wld^s^* mice [14], [19]. We therefore reasoned that any alterations to the proteome observed in *Wld^s^* mice at either 24 hrs or 48 hrs after injury were more likely to represent systemic responses to the lesion injury itself rather than changes directly related to the process of synaptic and axonal degeneration. iTRAQ proteomic analyses were therefore performed on tissue from *Wld^s^* mice using the same protocol adopted for our initial experiments on wild-type animals. Through subtracting candidates identified in synapse-enriched fractions from both wild-type and *Wld^s^* mice following injury (Table S2; validated using western blotting at 72 hrs after injury, Figure 1B), we refined our dataset to include only those putative proteins with modified expression in synapse-enriched fractions undergoing degeneration (Figure 1C–1D). This approach removed a total of 19 candidates from the dataset, resulting in a refined profile comprised of 93 putative proteins.

**Figure 2:**
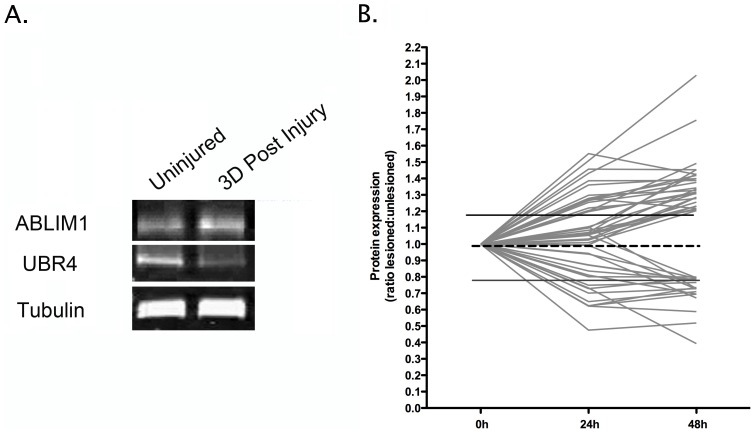
Temporal expression profiling identifies molecular changes occurring in synapse-enriched fractions from the striatum undergoing degeneration. A. Representative bands from fluorescent western blots for one up-regulated protein (Ablim1) and one down-regulated protein (Ubr4) in degenerating synapse-enriched fractions, validating expression changes observed in proteomic experiments. Tubulin is shown as a loading control. B. Graphical representation of protein expression changes for all 47 proteins modified in degenerating synapse-enriched fractions (see Table 1), illustrating global trends in the magnitude and scope of alterations identified.

Given that we planned to use data generated by our proteomics analysis to directly guide subsequent molecular genetic experiments in *Drosophila* (see below), we wanted to exclude any of the 93 putative proteins identified for which we could not be certain of their identity based on the peptide sequences reported from the proteomics analysis. We therefore excluded all putative proteins identified only by 1 unique peptide that we could not subsequently validate by western blotting. Examples of validation western blots for one up-regulated (ABLIM1) and one down-regulated (UBR4) protein are shown in Figure 2A. This generated a final refined list of 47 unique proteins with robustly modified expression in synapse-enriched fractions 48 hrs after injury (Table S3; Figure 2B). These 47 proteins were therefore considered to represent robust molecular perturbations occurring in synapse-enriched fractions during the initiation and onset phases of neurodegeneration *in vivo*.

### A complex temporal profile of protein expression changes during neurodegeneration

Expression mapping of all 47 proteins identified as having modified expression levels in degenerating synapse-enriched fractions allowed them to be grouped according to the temporal dynamics of their responses, as well as magnitude of expression change (Figure 2B). Given that synaptic degeneration was absent in the striatum 24 hrs after cortical lesion, but was widespread at 48 hrs after lesion [14], [19], we reasoned that individual proteins responding within 24 hrs of lesion were more likely to represent immediate-early responders and initiators of the degeneration process. By contrast, we reasoned that individual proteins whose expression levels were found to be altered only at 48 hrs after the lesion were more likely to represent effector pathways involved with the onset of degeneration.

**Figure 3:**
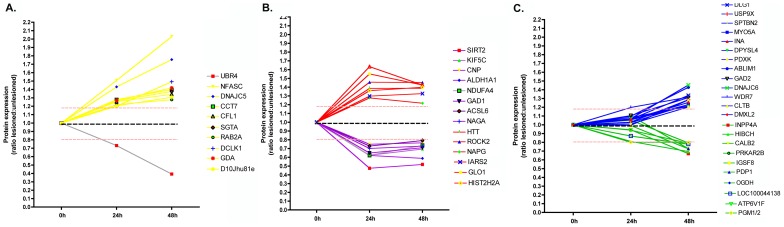
Temporal expression profiling for individual proteins identified in synapse-enriched fractions undergoing degeneration. Temporal profiles of protein expression changes in degenerating synapse-enriched fractions were grouped into 3 distinct categories: proteins with expression changes >20% by 24 hours, with further progressive alterations by 48 hours (A); proteins up or down regulated >20% by 24 hours following injury but with no subsequent increase/decrease (B); and proteins not changed at 24 hours but up or down regulated >20% at 48 hours following injury (C). Proteins responding within 24 hrs of lesion were considered to represent immediate-early responders and initiators of the degeneration process, whereas proteins whose expression levels were found to be altered only at 48 hrs after the lesion were considered to represent effector pathways involved with the onset of degeneration.

Diverse temporal patterns of expression changes were observed across the 47 proteins examined, suggesting that our analyses had detected proteins contributing both to early initiating phases and onset phases of degeneration (Figure 3). Of the 47 synaptic proteins identified 24 responded within 24 hrs of injury. Of these, 14 remained stable at 48 hrs whereas 10 showed additional incremental changes by 48 hrs. The other 23 proteins were unchanged at 24 hrs, responding only at 48 hrs after injury (Figure 3).

### *In silico* analysis revealed significant functional clustering of proteins

Next, we wanted to establish whether the profile of individual protein alterations identified in degenerating synapse-enriched fractions represented perturbations of specific functional pathways. We performed an *in silico* systems level analysis on the proteomics data using Ingenuity Pathway Analysis (IPA) software. This analysis identifies statistically significant functional clustering of proteins, based on known protein interactions and biological functions reported in the published literature [18]. Functional networks identified by the IPA software are statistically ranked according to a score calculated via a right-tailed Fischer's exact test, taking into account the number of original input proteins and the size of the network generated as a result. Only networks comprised of 3 or more identified proteins and reported with a *P* value of <0.05 were considered as being significant. These experiments revealed that the 47 identified proteins were functionally clustered into a relatively small group of networks (Table S4), focused principally around pathways regulating synaptic function (including synaptic transmission, exocytosis, transport of vesicles and formation of vesicles) and neurite development (including guidance of axons, formation of filaments, development of neurites and biogenesis of the cytoskeleton).

The *in silico* analysis also highlighted many proteins previously implicated in molecular pathways underlying neurological conditions (Table S4). Interestingly, these included neurodegenerative conditions where synapses and axons are known to be primary pathological targets (e.g. Alzheimer's disease, Parkinson's disease and HD; see introduction).

### Molecular pathways underlying synapse pathology are conserved from injury to disease

Next, we wanted to establish whether molecular pathways modified as a result of injury-induced degeneration were similarly modified in synapse-enriched fractions undergoing pathological alterations in neurodegenerative diseases resulting from genetic mutations. We therefore selected 11 proteins from our injury proteomics data where reliable antibodies were available for use in quantitative fluorescent western blotting experiments (ABLIM1, SPBTN, CCT7/TCP1, CFL1, CNP, DNAJC5/CSP, INPP4A, NFASC, ROCK2, SIRT2 and UBR4). These included candidates from the top five cellular process categories identified in our functional clustering analysis (see Table S4). We quantified expression levels in synapse-enriched fractions isolated from two distinct mouse models of neurodegenerative disease at early-symptomatic time-points: a genetic disease model with synaptic degeneration (the R6/2 mouse model of HD) and a model of spinocerebellar ataxia type 5 with synaptic dysfunction/dysregulation (βIII-spectrin knockout mouse).

**Figure 4:**
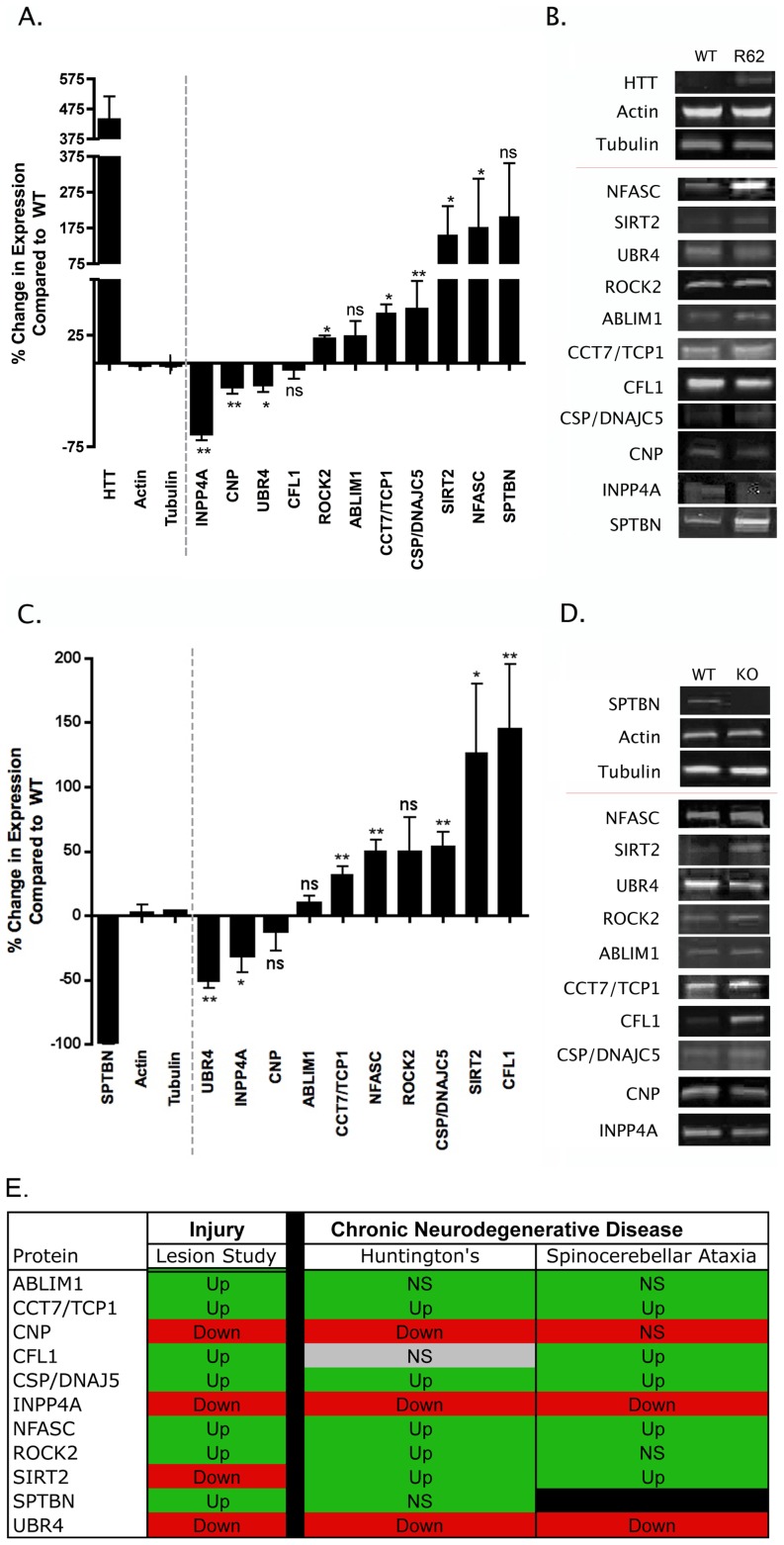
Molecular pathways underlying degeneration in synapse-enriched fractions are conserved across diverse neurodegenerative conditions. A. Bar chart (mean±SEM) showing quantitative fluorescent western blot data for protein expression levels in synapse-enriched fractions from the striatum generated from the R6/2 transgenic mouse model of HD compared to wild-type controls (N≥3 mice per genotype). Bars to the left of the dotted line show levels of control proteins (including Huntingtin [HTT]). Bars to the right of the dotted line show levels of proteins previously identified following injury (see Table 1). NS = not significant; *P<0.05; **P<0.01; Mann-Whitney test. B. Representative bands from quantitative fluorescent western blot experiments on synapse-enriched fractions from wild-type (WT) and R6/2 mice. C. Bar chart (mean±SEM) showing quantitative fluorescent western blot data for protein expression levels in synapse-enriched fractions from the cerebellum generated from a mouse model of spinocerebellar ataxia type 5 (βIII-spectrin KO mice) compared to wild-type controls (N≥3 mice per genotype). Bars to the left of the dotted line show levels of control proteins (including βIII-spectrin [SPTBN]). Bars to the right of the dotted line show levels of proteins previously identified following injury (see Table 1). NS = not significant; *P<0.05; **P<0.01; Mann-Whitney test. D. Representative bands from quantitative fluorescent western blot experiments on synapse-enriched fractions from wild-type (WT) and βIII-spectrin mice (KO). E. Comparison of protein expression changes in synapse-enriched fractions from the injury model (summarized from the proteomics data), R6/2 mice and βIII-spectrin KO mice. Red boxes indicate mean expression decreased >10%, green boxes indicate mean expression increased >10%, grey boxes indicate changes <10%. Up = significantly upregulated compared to controls; Down = significantly downregulated compared to controls; NS = not significantly changed compared to controls.

The pathophysiology of HD involves aggregation of mutated huntingtin (Htt) protein, transcriptional dysregulation, altered energy metabolism, excitotoxicity, impaired axonal transport and synaptic pathology [20]. The R6/2 mouse model of HD exhibits a progressive and fatal neurological phenotype, with synaptic alterations notable in the striatum [4]. Protein expression levels were analysed in synapse-enriched fractions generated from the striatum of R6/2 mice carrying a CAG repeat of 259–266 at 9–10 weeks of age (representing early-symptomatic stages of the disease). Of the 11 proteins examined, 8 showed significant changes in expression levels in fractions prepared from R6/2 mice (Figure 4A and 4B).

The βIII-spectrin knockout mouse models many of the human aspects of spinocerebellar ataxia type 5, including; synaptic dysfunction, postural abnormalities, progressive loss of motor coordination, and cerebellar degeneration [5]. Protein expression levels were analysed in synapse-enriched fractions generated from the cerebellum of βIII-spectrin knockout mice at 12 weeks of age (representing early-symptomatic stages of the disease [5]). Of the 10 proteins examined (SPTBN is knocked out in these mice), 7 showed significant changes in expression levels in βIII-spectrin knockout mice (Figure 4C and 4D).

Comparisons of protein expression data obtained from the cortical lesion model, R6/2 model and βIII-spectrin knockout revealed that 9 of the examined proteins showed expression changes occurring in the same direction across all three models (Figure 4E). Although the magnitude of identified expression changes were not always identical between models (and often were variable between individual mice), this likely represents the differing extent and nature of synaptic pathology observed between the three models at the time-points examined [4], [5], [14].

### Identification of individual proteins capable of independently regulating synapse and distal axon degeneration *in vivo*

Although we had obtained a clear understanding of conserved molecular alterations occurring in synapse-enriched fractions undergoing neurodegeneration, it remained unclear whether or not any of the proteins and pathways identified were capable of actively modulating synaptic and axonal stability and degeneration *in vivo*. We therefore used a molecular genetic approach in *Drosophila* to screen individual proteins for a direct role in neurodegeneration.

**Figure 5:**
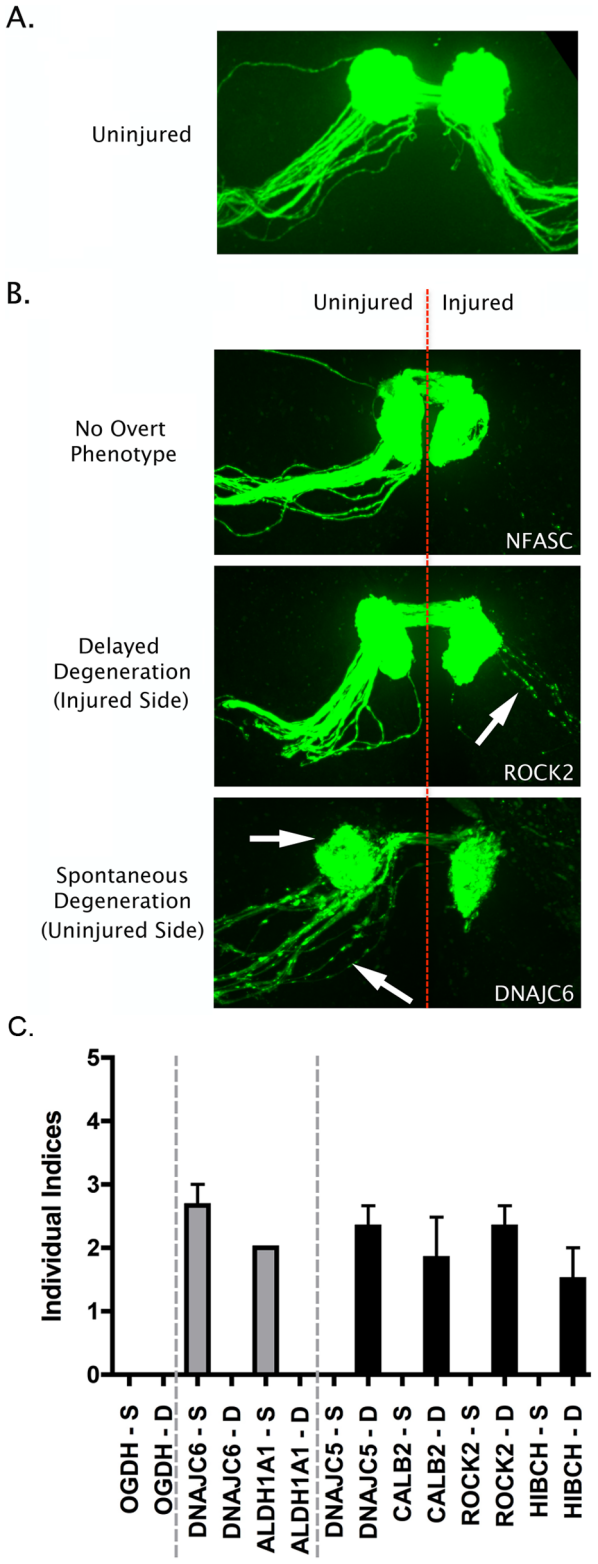
Overview of putative axo-synaptic degeneration phenotypes observed in *Drosophila* neurodegeneration screens. A. Representative confocal micrograph showing the morphology of the intact *Drosophila* olfactory receptor neuron (ORN) system, with axons and synaptic fields labeled with GFP in the *UAS-mCD8::GFP,OR22a-Gal4/+* background. Axons enter the antennal lobe laterally and project medially across the lobe to reach their target glomerulus, where synapses are located (see reference [21]). B. Representative confocal micrographs showing three distinct phenotypic profiles observed in injured and un-injured ORN axons and synapses 7 days after unilateral (right hand side of image) antennal ablation. The top panel shows intact healthy axons and synapses on the uninjured side and complete axonal degeneration (indicated by absence of GFP labeled profiles) on the injured side (example from an NFASC mutant). The middle panel shows delayed axo-synaptic degeneration on the injured side, as indicated by the retention of GFP-labelled axon profiles 7 days after injury (white arrow; example from a ROCK2 mutant). The bottom panel shows spontaneous (i.e. not injury-induced) axo-synaptic degeneration in the uninjured axons and synapses, indicated by reduction and fragmentation of GFP labeled axons and synapses (white arrows; example from a DNAJC6 mutant). C. Bar chart (mean±SEM) showing index scores (see methods) for spontaneous degeneration (S; grey bars) and delayed degeneration (D; black bars) in 7 mutant *Drosophila* lines. OGDH is shown as an example of a mutant line with no overt phenotype. DNAJC6 and ALDH1A1 mutants revealed evidence for spontaneous degeneration in the absence of any injury stimulus. DNAJC5, CALB2, ROCK2 and HIBCH mutants revealed evidence for delayed degeneration following antennal ablation.

We examined the role of individual proteins in regulating synaptic stability and degeneration using the *Drosophila* olfactory system to screen a collection of existing mutants, or lines with transposon insertions in a subset of these genes. Briefly, mutants and insertion lines were crossed in to a background that allowed visualization of a subset of olfactory receptor neurons (ORNs; OR22a-Gal4/UAS-mCD8::GFP). Distal axons and their synaptic fields in the antennal lobe were examined in uninjured controls as well as 7 days after surgical ablation of antennae. Examining uninjured controls allowed us to screen individual mutant lines and test whether they modified basal synaptic and axonal stability (e.g. do synapses and axons degenerate spontaneously in the mutant line?). Spontaneous degeneration was identified by the presence of fragmented axons and absence/decrease of GFP signal in the glomeruli housing synaptic terminals of ORNs [21] and scored using a spontaneous degeneration index, where a score of 0 represented no disruption of axons or synapses in the glomerulus and 5 indicated complete spontaneous breakdown (see methods; Figure 5). Surgical ablation of antennae triggered rapid axonal and synaptic degeneration, which is complete within one day in wild-type controls, and axonal debris is cleared within one week after injury [21]. Screening individual mutant lines 7 days after surgical ablation therefore allowed us to examine whether any of the mutations resulted in a delay in the rate of injury-induced degeneration, scored using a delayed degeneration index where a score of 0 indicated no delay in degeneration and 5 indicated a complete block (see methods; Figure 5).

From our original list of 47 synaptic proteins we obtained *Drosophila* lines for 21 different genes that harbored either defined mutations known to affect that gene, or P element insertions within the locus identified by the *Drosophila* Genome Project (see methods). Of the 34 mutant lines obtained, 14 produced viable flies suitable for analyses of axonal and synaptic stability and degeneration (covering a total of 13 individual proteins; Table 1). Eight of the lines examined showed no overt phenotype in either stability or degeneration assays (Table 1). However, 6 mutant lines were found to independently modulate stability or degeneration of distal axons and synapses in ORNs. Mutants of both ALDHA1 and DNAJC6/Auxillin caused spontaneous degeneration of distal axons and synaptic terminals in uninjured ORNs (Figure 5). In contrast, mutations affecting CALB2/Calretinin, DNAJC5/CSP, HIBCH and ROCK2 caused a partial delay of injury-induced degeneration of axons and synapses (Figure 5). In each of these lines, intact distal axons or axonal fragments were observed 7 days after experimental nerve lesion, a time-point at which axonal remnants were never observed in wild-type flies (data not shown [21]).

**Table 1:** List of viable *Drosophila* lines tested in the current study.

Protein	Bloomington ID	Observation
ALDHA1	12900	Spontaneous Degeneration
Auxillin/DNAJC6	26277	Spontaneous Degeneration
CALB2/calretinin	18382	Delayed Degeneration
CFL1	7762	No Overt Phenotype
CSP/DNAJC5	20497	Delayed Degeneration
DLG1	12301	No Overt Phenotype
HIBCH	30075	Delayed Degeneration
HTT	24665	No Overt Phenotype
INPP4A	18046	No Overt Phenotype
NFASC	5595	No Overt Phenotype
OGDH	23173	No Overt Phenotype
ROCK2	6671	Delayed Degeneration
VPS29	13491, 20672	No Overt Phenotype

**Figure 6:**
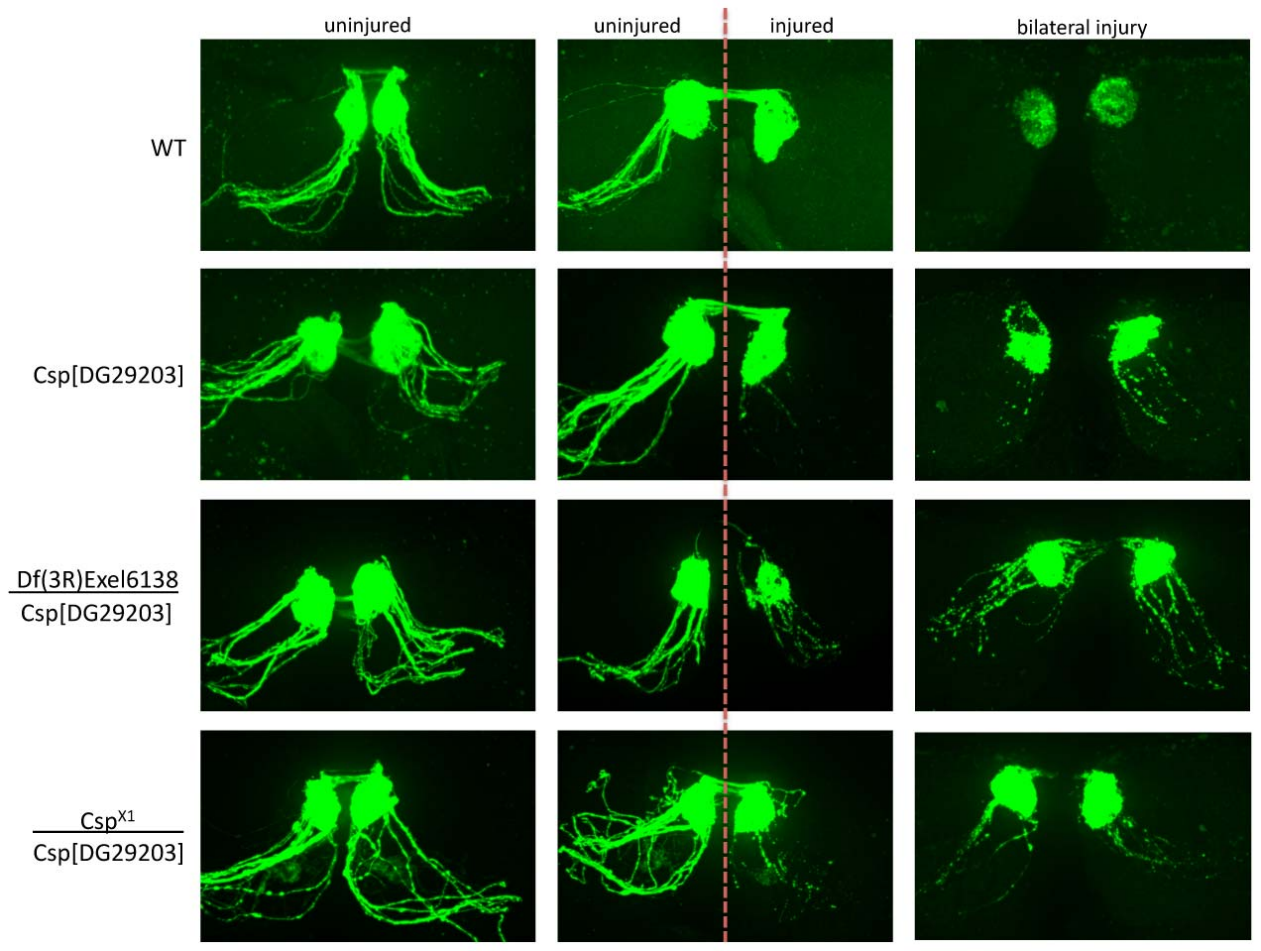
Detailed genetic analysis confirms DNAJC5/CSP as a robust regulator of axo-synaptic degeneration *in vivo.* Representative confocal micrographs showing axon degeneration profiles in wild-type (WT) flies and additional DNAJC5/CSP lines: *csp^X1^*, a loss of function allele which deletes the first exon of *csp*; and *Df(3R)Exel6138*, a deletion which completely removes the *csp* locus. Examples are shown of uninjured axons (left panels), unilaterally injured axons (middle panels) and bilaterally injured axons (right panels). Both *csp^X1^* and *Df(3R)Exel6138* failed to complement the delay in axonal degeneration observed with our original allele (*csp^DG29203^*), thereby mapping this phenotype to the *csp* locus. Note how the severity of the delay in axonal degeneration was enhanced when *csp^DG29203^* was placed over either of these null alleles of *csp*, suggesting that *csp^DG29203^* is a weak loss of function allele.

To provide more robust genetic evidence for a role for one of these proteins (DNAJC5/CSP) in axonal and synaptic degeneration, we obtained two additional alleles: *csp^X1^*, a loss of function allele which deletes the first exon of *csp*; and *Df(3R)Exel6138*, a deletion which completely removes the *csp* locus. Both *csp^X1^* and *Df(3R)Exel6138* failed to complement the delay in axonal degeneration observed with our original allele (*csp^DG29203^*), thereby mapping this phenotype to the *csp* locus (Figure 6). We further note that the severity of the delay in axonal degeneration appeared to be enhanced when *csp^DG29203^* was placed over either of these null alleles of *csp*, which argues that *csp^DG29203^* is a weak loss of function allele.

Thus, several individual proteins initially identified as a result of having modified expression levels in synapse-enriched fractions undergoing neurodegeneration appear capable of directly influencing synaptic and axonal stability and degeneration in *Drosophila*. In addition, our work rigorously defines the role of DNAJC5/CSP as an *in vivo* regulator of synaptic and axonal degeneration.

## Discussion

In this study we report on the effective use of a novel ‘top-down’ approach for identifying individual proteins and functional pathways responsible for regulating neurodegeneration in synaptic and axonal compartments of neurons. By undertaking a series of comparative quantitative proteomic screens on degenerating synapse-enriched fractions isolated from the mouse brain we identified 47 proteins with robustly modified expression levels during the early stages of neurodegeneration. We showed that molecular responses to degeneration occurring in synapse-enriched fractions following injury were recapitulated in synapse-enriched fractions undergoing pathological changes as a result of disease-causing genetic mutations. We also used our proteomic data to design molecular genetic screens in *Drosophila* that revealed roles for 6 proteins in regulating synaptic and axonal degeneration *in vivo*. These findings further our understanding of mechanisms regulating the active degeneration of synapses and axons, providing a basis from which to develop novel neuroprotective strategies for a range of neurodegenerative conditions.

An initial comparison of the 6 individual proteins found to directly mediate synaptic and axonal stability and degeneration in our *Drosophila* screen reveals a diverse range of biological functions. For example, both DNAJC5/CSP and DNAJC6 belong to the evolutionarily conserved DNAJ/HSP40 family of proteins that regulate molecular chaperone activity by stimulating ATPase activity [22], whereas CALB2/calretinin is an intracellular calcium-binding protein [23] and ROCK2 is a Rho kinase belonging to a family of serine/threonine kinases involved in structural remodeling of the cytoskeleton [24]. Despite this apparent heterogeneity, it should be noted that the *in silico* analysis of data generated by our proteomics experiments highlighted significant clustering of proteins within functional networks that regulate synaptic transmission. This finding is further reinforced by comparisons of the biological roles of the 6 proteins found to independently regulate degeneration in our *Drosophila* screen, 5 of which have been implicated in the control of synaptic function: both CALB2/Calretinin and ALDHA1 modulate synaptic long-term potentiation (LTP) [25], [26], DNAJC6 has been implicated in clatherin-mediated synaptic vesicle recycling [27], [28], DNAJC5/CSP plays a role in SNARE-complex assembly [29], and ROCK2 levels influence synaptic transmission and plasticity [30]. Taken together with previous reports linking perturbations in synaptic transmission with synaptic degeneration [11] (also see below), our findings suggest that endogenous neuronal proteins and pathways regulating synaptic function play an important role in modulating neurodegenerative pathways.

It is worth noting, however, that at least one of the other proteins found to influence degeneration in our *Drosophila* screen (HIBCH; 3-hydroxyisobutyryl-CoA hydrolase) is unlikely to impact directly on synaptic transmission pathways. HIBCH plays an important role in valine catabolism, disruption of which is sufficient to induce progressive infantile neurodegeneration in humans [31]. Thus, multiple cellular and molecular pathways are likely to converge on mechanisms regulating synaptic and axonal degeneration. This finding is supported by our *in silico* analysis revealing that several of the proteins identified in our screen also contribute to pathways regulating neurite development. This supports previous observations from *Drosophila* models linking ubiquitin-mediated developmental processes with neurodegenerative processes occurring in axonal compartments of neurons [32]. Thus, although proteins and pathways involved in synaptic transmission are likely to contribute significantly to neurodegeneration, other distinct molecular pathways also appear to be capable of influencing synaptic and axonal degeneration *in vivo*.

Only one of proteins we identified as a direct mediator of degeneration, DNAJC5/CSP, belongs to the small group of endogenous genes and proteins previously reported to directly affect synaptic stability and degeneration *in vivo*. DNAJC5/CSP has been implicated in synaptic degeneration contributing to the pathogenesis of neurodegenerative diseases [11], [33]. However, our findings are partially inconsistent with previously published studies examining the role of DNAJC5/CSP in animal models. For example, Fernández-Chacón and colleagues reported that loss of CSP expression in mice caused synaptic degeneration in the CNS, leading them to conclude that increased levels of the protein may be neuroprotective [15], [34]. By contrast, we found that DNAJC5/CSP levels are robustly and consistently *increased* in degenerating synapse-enriched fractions following injury and in synapse-enriched fractions from mouse models of neurodegenerative disease. Moreover, a thorough genetic analysis in *Drosophila* using well-defined mutants in DNAJC5/CSP revealed that loss of CSP is neuroprotective, delaying degeneration in axonal and synaptic compartments. Thus, whilst it is clear that DNAJC5/CSP needs to be regarded as a critical regulator of-neuronal stability and degeneration *in vivo*, precise details correlating expression levels with its role in stabilizing distal axons and synapses during disease-induced degeneration remain to be determined.

Given that only partial coverage of the entire synaptic proteome is possible through the coupling of subcellular fractionation with current proteomics technologies, alongside the stringent 20% cut off threshold employed, the refinement methodologies applied in the current study and the limited number of viable fly lines that we screened, it is highly likely that additional genes and proteins capable of regulating neurodegeneration remain to be discovered. Our uncovering of molecular responses underlying neurodegeneration in distal compartments of neurons, alongside the identification of 5 novel mediators of degeneration and new experimental insights into the role of DNAJC5/CSP, suggests that combining proteomic screens on synapse-enriched fractions with axonal/synaptic degeneration assays in *Drosophila* provides a powerful approach for elucidating mechanisms of neurodegeneration *in vivo*.

## Materials and Methods

### Ethics statement

All animal experiments were approved by a University of Edinburgh internal ethics committee and were performed under license by the UK Home Office (project license number 60/3891).

### Mouse cortical lesion model

Two month old, female C57Bl/6 (wild-type) and *Wld^s^* mice were obtained from Harlan Olac Laboratories (Bicester, UK) and housed within the animal care facilities in Edinburgh. Care was taken to ensure that the wild-type mice did not contain the alpha-synuclein gene deletion that was present in a sub-strain of Harlan Olac Bl6 mice [18]. All surgical procedures were performed under license from the UK Home Office. General anaesthesia was induced using a mixture of isopentane and oxygen, before securing the head in a Kopf stereotaxic frame. Fur overlying the cranial vault was shaved with scissors before making an incision through the skin at the midline. Four holes were drilled on the left side of skull; 1) in the midline at bregma, 2) in line with the first but at the level of lambda, 3) further caudal on the lateral side just above the temporalis muscle, 4) anterolateral in line with the first and third holes. The skull was cut in lines connecting all holes except the most caudal border, and then reflected. A suction pipette was used to remove all visible cortex under a dissecting microscope, down to the level of the corpus callosum, before replacing the skull-flap [14], [19]. The lesion site was filled with gel foam (Ethicon) before replacing the skull-flap. Overlying skin was then sutured and the mouse placed on a heated blanket until recovered fully from the anaesthetic. Mice were maintained in standard animal house conditions and were checked daily for any signs of distress or discomfort as previously described [14], [19].

### Mouse disease models

R62 mice with a CAG repeat number of 259–266 from breeding colonies at the University of Cambridge were sacrificed at 9–10 weeks old. Mice were genotyped as previously described [20]. Mice lacking βIII-spectrin and age-matched controls from breeding colonies at the University of Edinburgh were raised and sacrificed at 12–15 weeks old. Mice were genotyped as previously described [5].

### Preparation of synapse-enriched fractions

Brains were rapidly removed following sacrifice and required brain regions microdissected out (cerebellum from βIII-spectrin mice, striatum from wild-type mice, *Wld^s^* mice subjected to a cortical lesion and R6/2 mice). Synapse-enriched fractions were prepared as previously described [18]. Briefly, brain regions were homogenised in an ice-cold isotonic sucrose solution (0.32 M sucrose, 1 mM EDTA, 5 mM Tris-HCl, pH 7.4). Homogenate was centrifuged in a fixed-angle rotor at 900 *g* for 10 min and the supernatant (S1) was collected. The pellet (P1) was resuspended in sucrose solution and centrifuged again at 900 *g* for 10 min. The resulting supernatant (S1′) was combined with S1 and centrifuged in a fixed angle rotor at 20,000 g for 15 min. The supernatant (S2) was discarded and the pellet (P2) containing crude synapse-enriched fractions was washed in a Krebs-like buffer (118.5 mM NaCl, 4.7 mM KCl, 1.18 mM MgCl_2_, 0.1 mM K_2_HPO_4_, 20 mM Hepes, 1.3 mM CaCl_2_, 10 mM glucose, pH 7.4) then centrifuged at 14,000 *g* for 10 min.

### Quantitative Western blots

Quantitative fluorescent western blotting was performed as previously described [35]. Briefly, protein was extracted (N>3 mice per sample) in RIPA buffer with 10% protease inhibitor cocktail (Sigma). 15–30 µg of protein per lane was separated by SDS/Polyacrylamide gel electrophoresis on 4–20% pre-cast NuPage 4–12% Bis Tris gradient gels (Invitrogen) and then transferred to PVDF membrane overnight. The membranes were then blocked using Odyssey blocking buffer (Li-COR) and incubated with primary antibodies as per manufacturers instructions (ABLIM1, SPBTN, CCT7/TCP1, UBR4 - Santa Cruz; Beta-actin, BIII-tubulin, CNP, CFL1, CSP, DOCK7, HTT, INPP4A, NFASC, ROCK2, SIRT2, VAT1 - Abcam). Odyssey secondary antibodies were added according to manufacturers instructions (Goat anti rabbit IRDye 680 and Goat anti mouse IRDye 800). Blots were imaged using an Odyssey Infrared Imaging System (Li-COR Biosciences). Scan resolution of the instrument ranges from 21–339 µm and in this study blots were imaged at 169 µm.

### iTRAQ proteomics

Protein was extracted from synapse-enriched fractions in MEBC buffer (50 mM Tris, 100 mM NaCl, 5 mM NaEDTA, 5 mM NaEGTA, 40 mM glycerophosphate, 100 mM NAF, 100 mM Sodium orthovanadate, 0.25% NP40, 1 Roche “complete” protease inhibitor tablet, pH 7.4) before acetone precipitation and labeling for iTRAQ analysis as previously described [35]. Samples (N = 36 mice in total. N = 18 mice per genotype, N = 6 mice per time point) were precipitated with −20°C chilled acetone (1∶4, vol/vol) and stored at −20°C overnight. The precipitates were spun at 4°C for 10 min then washed with an acetone∶water mixture (4∶1, vol/vol) twice prior to air drying. The pellets were then re-suspended in iTRAQ sample buffer (25 µl 500 mM TEAB, 1 µl denaturant (2% SDS) and 2 µl of reducing agent (TCEP)). The samples were allowed to incubate for 1 hour at 60°C prior to protein estimation in triplicate (3×1 µl) by microBCA assay (Pierce).

Aliquots of each sample equivalent to 100 µg were made up to 28 µl using the iTRAQ sample buffer minus denaturant. To each sample 1 µl of 84 mM IAA was added, the samples mixed and spun prior to incubation at room temperature in the dark for 30 minutes. To each sample 10 µl of a 1 µl/µl solution of trypsin (Sequencing grade, Roche) in water was added and the samples incubated overnight on a shaking platform at 30°C. To each vial of iTRAQ reagent (113, 114, 115, 116, 117, 118) 70 µl of ethanol was added, mixed and spun prior to transfer to each sample vial (WT0 hrs-114, WT24 hrs-116, WT48 hrs-118; Wlds0 hrs-113, Wlds24 hrs-115, Wlds48 hrs-117). The pH was checked for each sample to ensure pH was greater than 8.0 prior to incubation for 1 hour at room temperature. 100 µl of water was added to each sample to quench the reaction prior to pooling of the six iTRAQ labelled samples and subsequent drying by vacuum centrifugation as previously described [35].

The pooled iTRAQ sample was resuspended in 50 µl of 25% acetonitrile in 0.1% formic acid prior to loading through a home made ziptip using 10 µl of 10% slurry of Poros HS in 50∶50 methanol∶water. The ziptip was then washed with 3×25 µl of 25% acetonitrile in 0.1% formic acid prior to loading of the pooled iTRAQ sample. The ziptip was then washed with 3×25 µl of 25% acetonitrile in 0.1% formic acid prior to elution with a stepped NaCl gradient in 25% acetonitrile in 0.1% formic acid. A fraction of iTRAQ labelled peptides were then eluted with 2×25 µl of 5–800 mM NaCl (5, 10, 20, 50, 100,150, 200, 150, 300, 400, 800) in 25% acetonitrile in 0.1% formic acid. A final elution of the ziptip with 200 mM NH4OH and 50% propanol was used to remove the most hydrophobic peptides. Each fraction was then dried by vacuum centrifugation and stored until mass spectrometry analysis of pooled iTRAQ samples by nano liquid chromatography-mass spectrometry/mass spectrometry (nLC-MS/MS). Prior to the analysis, each dried SCX fraction was re-suspended in 35 ml of 1% formic acid and 10 ml aliquots were injected onto an Agilent 6520 Q-TOF using an Agilent 1200 series nanoLC system with microfluidic interface as previously described [35].

Raw data files were converted to mascot generic file (mgf) by MassHunter workstation software prior to merging of the files with Mascot Daemon and subsequent database (IPI Mouse) searching with the Mascot search engine (Matrix Science, Version 2.2). To be considered as a protein with modified relative expression, the peptide abundance (or average of all peptide abundances for proteins identified by more than one unique peptide) had to be modified by greater than 20% (up or down) [35]. For validation, expression levels of a number of proteins which were identified by a single peptide were quantified in freshly prepared tissue samples using quantitative fluorescent western blotting (see above).

Ingenuity Pathways Analysis (IPA) statistical network analyses were performed as previously described [18].

### *Drosophila* degeneration screen

*Drosophila* orthologs of the designated mouse proteins were identified, when available, using a reciprocal BLASTing approach and the Ensembl website (ensemble.org). Available mutations were identified in Flybase the following *Drosophila* mutant stocks were obtained from the Bloomington Stock Center (Bloomington ID #): 12900, 26277, 18382, 7762, 20497, 12301, 30075, 24665, 18046, 5595, 23173, 6671, 13491, 20672, 25213, 18502, 13446, 9109, 11876, 7084, 25107, 7938, 5708, 11734, 29228, 2247, 13491, 15889, 8479, 18884, 23097, 15642, 382, 32035, and 7617. Mutant stocks on the X chromosome were crossed to *OR22a-Gal4, UAS-mCD8::GFP* flies and males were used for the degeneration screen. Stocks for mutants on the second chromosome were crossed to *Sp/Cyo*; *OR22a-Gal4, UAS-mCD8::GFP*, and the progeny were self-crossed to obtain homozygous mutant flies on the second and *OR22a-Gal4, UAS-mCD8::GFP* on the third chromosome. Similarly, stocks for mutants on the third chromosome were crossed to *OR22a-Gal4, UAS-mCD8::GFP*; *Dr/TM3* and the progeny self-crossed to obtain homozygous mutant flies on the third chromosome and *OR22a-Gal4, UAS-mCD8::GFP* on the second chromosome.

The Wallerian degeneration assay was performed as described [21]. Briefly, flies were aged for 7 days after eclosion to allow strong labeling of ORN axons with GFP. The left 3^rd^ antennal segment was then removed, and the flies aged for another 7 days. The right antennal ORNs served as unlesioned controls. The fly heads were then fixed in 4% formaldehyde/PBS/0.1% Tween, and dissected. Fly brains were mounted in Vectashield and examined by confocal microscopy where they were phenotypically scored by an investigator who was unaware of the genotype. A minimum of 10 animals were examined and assessed per mutant strain.

Spontaneous degeneration in uninjured axons and synapses was scored by eye using the following criteria: 0 = no evidence for fragmentation of axons or loss of GFP fluorescence in the glomerulus; 1 = <10% of axons showing fragmentation and/or mild loss of GFP fluorescence in the glomerulus; 2 = <25% of axons showing fragmentation and/or mild to moderate loss of GFP fluorescence in the glomerulus; 3 = >50% of axons showing fragmentation and/or moderate to severe loss of GFP fluorescence in the glomerulus; 4 = only fragmented axons remaining and/or severe loss of GFP fluorescence in the glomerulus; 5 = no GFP signal remaining in axons or the glomerulus. Delayed degeneration in injured axons and synapses was scored by eye using the following criteria: 0 = no intact axons and no fragments remaining; 1 = no intact axons but fragmented debris remaining; 2 = ∼25% of axons intact but with extensive fragmentation of surrounding axons; 3 = ∼50% of axons intact with evidence for fragmentation in surrounding axons; 4 = >75% of axons intact with only modest amounts of fragmentation in surrounding axons; 5 = intact axons and synapses with no evidence for fragmentation.

### Statistical analysis

Statistical analyses were performed using either Ingenuity Pathways Analysis (IPA) software (for analysis of proteomic data) or GraphPad Prism software (for data from quantitative western blots). *P*<0.05 was considered to be statistically significant.

## Supporting Information

Table S1 All proteins with altered expression levels >20% in wild-type striatal synaptosome preparations at 24 and/or 48 hrs after cortical lesion.
(PDF)

Table S2 All proteins with altered expression levels >20% in Wld^S^ striatal synaptosome preparations at 24 and/or 48 hrs after cortical lesion.
(PDF)

Table S3 Proteins with altered expression levels >20% in striatal synapse-enriched preparations from wild-type mice 48 hrs after cortical lesion (emPAI = exponentially modified protein abundance index).
(PDF)

Table S4 Systems level analysis of functional clustering of proteins modified in degenerating synapses (networks identified as statistically significant clusters [P<0.05], IPA analysis).
(PDF)
