# Supplementary material for: Combining Comparative Proteomics and Molecular Genetics Uncovers Regulators of Synaptic and Axonal Stability and Degeneration In Vivo
Source: PLoS Genet. 2012 Aug 30;8(8):e1002936. doi: 10.1371/journal.pgen.1002936 (PMC3431337; doi:10.1371/journal.pgen.1002936)
Supplement: Table S1 — All proteins with altered expression levels >20% in wild-type striatal synaptosome preparations at 24 and/or 48 hrs after cortical lesion. (PDF) [file pgen.1002936.s001.pdf]

Table S1: All proteins with altered expression levels >20% in wild-type striatal synaptosome preparations at 24 and/or 48hrs after cortical lesion

Accession Number	Symbol	Protein Name	Mol Weight (Da)	Peptides	Score	emPAI	Average Ratio 24Hrs vs Unlesioned	Average Ratio 48Hrs vs Unlesioned
IPI00329927.4	NFASC	Neurofascin	155723	34	51.2	0.04	1.508	2.030
IPI00751955.1	TMX2	Putative Uncharacterized Protein Tmx2	35998	17	179.4	0.09	1.623	1.913
IPI00875866.1	DNAJC5	23 Kda Protein	28769	15	384.7	0.25	1.431	1.756
IPI00187545.4	PLXNA4	Plexin-A4	250164	54	85.9	0.01	1.464	1.694
IPI00468380.4	DCLK1	Isoform 1 of Serine/Threonine-Protein Kinase Dclk1	100794	30	79.2	0.07	1.202	1.492
IPI00762713.1	DNAJC6	Isoform 3 of Putative Tyrosine-Protein Phosphatase Auxilin	123424	27	41.2	0.08	1.071	1.454
IPI00108150.1	ROCK2	Rho-Associated Protein Kinase 2	206952	68	89.1	0.02	1.457	1.453
IPI00473728.1	RPL14	Putative Uncharacterized Protein	35236	14	67.0	0.09	1.153	1.453
IPI00467530.3	ABLIM1	Isoform 1 of Actin-Binding Lim Protein 1	115052	29	61.7	0.03	1.098	1.428
IPI00623506.3	ANK3	Brain-Specific Ankyrin-G	577303	106	21.2	0.01	0.761	1.427
IPI00395042.1	NRCAM	Isoform 3 of Neuronal Cell Adhesion Molecule	162929	18	62.0	0.02	1.349	1.425
IPI00122399.1	GLG1	Golgi Apparatus Protein 1	164294	39	87.4	0.02	1.635	1.424
IPI00321734.7	GLO1	Lactoylglutathione Lyase	26747	11	112.5	0.42	1.552	1.424
IPI00469987.3	GDA	Guanine Deaminase	59707	39	66.9	0.17	1.269	1.419
IPI00126048.2	PSMD13	Isoform 1 of 26S Proteasome Non-Atpase Regulatory Subunit 13	50425	14	52.6	0.07	0.967	1.410
IPI00399905.5	PPFIA3	Liprin-Alpha-3	134308	52	79.4	0.02	1.451	1.409
IPI00331174.5	CCT7	T-Complex Protein 1 Subunit Eta	73208	81	89.0	0.09	1.279	1.400
IPI00272033.3	HIST2H2AC	Histone H2A Type 2-C	18543	9	170.1	0.95	1.360	1.399
IPI00222457.2	DARS	Aspartyl-Trna Synthetase, Cytoplasmic Isoform 1	66998	23	45.6	0.05	0.949	1.398
IPI00471246.2	IVD	Isovaleryl-Coa Dehydrogenase, Mitochondrial	54604	19	127.6	0.06	1.269	1.391
IPI00271166.4	HTT	Huntingtin	385122	111	66.6	0.01	1.386	1.388
IPI00116331.1	SGTA	Isoform 1 of Small Glutamine-Rich Tetratricopeptide Repeat-Containing Protein Alph	38788	15	114.3	0.28	1.266	1.376
IPI00153176.2	FBXO2	F-Box Only Protein 2	36678	5	56.8	0.09	0.576	1.376
IPI00265406.3	PFAS	Phosphoribosylformylglycinamidine Synthase	159634	27	137.1	0.02	2.063	1.365
IPI00226790.2	PIGT	Loc100046871 Gpi Transamidase Component Pig-T	73344	20	92.9	0.04	1.602	1.353
IPI00230449.7	HPCAL1	Hippocalcin-Like Protein 1	27914	17	119.0	0.40	1.114	1.346
IPI00890117.1	CFL1	Cofilin-1	26685	50	171.6	0.80	1.254	1.343
IPI00408668.2	DLG1	Loc100047603 Isoform 1 of Disks Large Homolog 1	117797	27	82.5	0.03	1.063	1.336
IPI00624192.3	DPYSL5	Dihydropyrimidinase-Related Protein 5	71782	32	161.4	0.09	1.128	1.328
IPI00453499.3	IARS2	Isoleucyl-Trna Synthetase, Mitochondrial	132429	30	88.5	0.02	1.298	1.327
IPI00337930.4	CKAP5	Isoform 1 of Cytoskeleton-Associated Protein 5	292550	73	257.7	0.02	1.538	1.317
IPI00653237.2	WDR7	Wdr7 Protein	183911	70	57.6	0.05	1.198	1.317
IPI00136716.1	GRM3	Metabotropic Glutamate Receptor 3	114621	20	35.7	0.06	1.283	1.316
IPI00318522.3	GAD2	Glutamate Decarboxylase 2	77901	44	226.7	0.28	1.106	1.311
IPI00133284.1	D10JHU81E	Loc100046684 Es1 Protein Homolog, Mitochondrial	34499	13	84.1	0.20	1.205	1.308
IPI00468688.4	TARS	Threonyl-Trna Synthetase, Cytoplasmic	103684	17	35.2	0.03	1.411	1.306
IPI00857896.1	6430548M08RIK	Hypothetical Protein Loc234797 Isoform B	54772	5	48.6	0.06	1.246	1.298
IPI00918347.1	PIP5K1C	76 Kda Protein	87979	30	80.1	0.08	1.162	1.292
IPI00126072.2	VAT1	Synaptic Vesicle Membrane Protein Vat-1 Homolog	49381	11	109.8	0.07	0.974	1.290
IPI00798468.1	USP9X	Ubiquitin Carboxyl-Terminal Hydrolase	334392	61	64.1	0.02	1.031	1.286
IPI00554923.1	CLTB	Isoform 2 of Clathrin Light Chain B	28446	15	40.8	0.39	0.995	1.285
IPI00137227.1	RAB2A	Ras-Related Protein Rab-2A	27658	10	98.8	0.26	1.220	1.278
IPI00675436.2	GM5422	Similar To Proteasome (Prosome, Macropain) 26S Subunit, Non-Atpase, 2 Isoform 1	108022	41	158.6	0.03	1.023	1.274
IPI00134353.3	NOL3	Nucleolar Protein 3	25693	6	87.4	0.13	1.393	1.258
IPI00896744.1	DMXL2	Dmx-Like Protein 2	392746	81	497.4	0.03	1.060	1.256
IPI00124291.1	VPS45	Vacuolar Protein Sorting-Associated Protein 45	77579	25	52.3	0.04	1.251	1.253
IPI00318614.9	IDH2	Isocitrate Dehydrogenase [Nadp], Mitochondrial	62890	28	59.2	0.05	1.277	1.249
IPI00221577.1	CPT1C	Carnitine O-Palmitoyltransferase 1, Brain Isoform	97634	19	151.1	0.03	0.957	1.249
IPI00465812.2	EPB4.1L1	Band 4.1-Like Protein 1 Isoform A	118655	39	37.8	0.03	1.097	1.248
IPI00225961.5	PHGDH	D-3-Phosphoglycerate Dehydrogenase	65257	23	95.8	0.05	1.155	1.237
IPI00120503.1	COPB1	Coatomer Subunit Beta	129432	47	41.7	0.03	0.902	1.237
IPI00223757.4	AKR1B3	Aldose Reductase	44265	13	34.4	0.07	1.527	1.232
IPI00134344.6	SPNB3	Putative Uncharacterized Protein	309135	78	83.1	0.05	1.025	1.232
IPI00118120.1	MYO5A	Myosin-Va	267915	102	198.8	0.08	1.065	1.231
IPI00881096.1	NAPG	Putative Uncharacterized Protein Napg	49418	34	206.7	0.38	1.278	1.217
IPI00135965.2	INA	Alpha-Internexin	63789	47	127.5	0.42	1.057	1.216
IPI00230566.4	GNG7	Guanine Nucleotide-Binding Protein G(I)/G(S)/G(O) Subunit Gamma-7	9849	17	106.7	0.35	0.831	1.216
IPI00313151.3	DPYSL4	Isoform 1 of Dihydropyrimidinase-Related Protein 4	72227	32	53.0	0.14	1.084	1.210
IPI00283511.1	PDXK	Pyridoxal Kinase	40753	40	261.7	0.73	1.009	1.204
IPI00116697.1	RAB6	Isoform 1 of Ras-Related Protein Rab-6A	27948	26	60.0	0.25	1.241	1.195
IPI00118900.1	PDE1B	Calcium/Calmodulin-Dependent 3',5'-Cyclic Nucleotide Phosphodiesterase 1B	71130	25	166.3	0.09	1.277	1.173
IPI00120342.6	SLC17A6	Vesicular Glutamate Transporter 2	74329	23	114.8	0.04	1.292	1.169
IPI00273164.1	ALDH5A1	Succinate-Semialdehyde Dehydrogenase, Mitochondrial	66542	43	126.4	0.21	1.245	1.168
IPI00381088.5	UNC13A	Putative Uncharacterized Protein Unc13A	242430	39	81.6	0.01	1.291	1.164
IPI00679051.1	CADPS2	Isoform 1 of Calcium-Dependent Secretion Activator 2	175106	69	44.9	0.04	1.449	1.145
IPI00123505.2	SYP	Synaptophysin	38546	53	152.5	0.78	1.265	1.141
IPI00626132.3	HSD17B10	Hydroxysteroid (17-Beta) Dehydrogenase 10	32729	37	433.1	0.34	1.316	1.137
IPI00115429.1	GGT7	Gamma-Glutamyltransferase 7	74611	23	187.8	0.04	1.285	1.137
IPI00228645.5	PFN2	Isoform 1 of Profilin-2	18406	18	50.6	0.66	1.320	1.135
IPI00108783.1	NF1	Isoform 2 of Neurofibromin	375725	44	158.5	0.01	1.489	1.131
IPI00955134.1	ARPC4	Putative Uncharacterized Protein	24068	54	76.1	0.68	1.273	1.129
IPI00116277.3	CCT4	T-Complex Protein 1 Subunit Delta	70711	34	804.5	0.15	1.316	1.125
IPI00129519.3	BASP1	Brain Acid Soluble Protein 1	30896	40	620.5	2.08	1.232	1.115
IPI00127707.1	PCBP2	Isoform 1 of Poly(Rc)-Binding Protein 2	44072	23	34.8	0.07	1.203	1.105
IPI00119663.3	MAPK1	Mitogen-Activated Protein Kinase 1	48949	49	119.8	0.48	1.223	1.097
IPI00118880.5	GUCY1B3	Guanylate Cyclase Soluble Subunit Beta-1	81390	28	70.2	0.08	1.201	1.093
IPI00112414.1	CSE1L	Exportin-2	129131	31	41.0	0.03	1.237	1.090
IPI00112001.1	CAP2	Adenylyl Cyclase-Associated Protein 2	63153	20	36.5	0.11	1.245	1.086
IPI00135677.2	EHD3	Eh Domain-Containing Protein 3	74833	28	128.2	0.09	1.809	1.080
IPI00308484.3	SPARCL1	Sparc-Like Protein 1	86814	24	40.2	0.04	1.268	1.076
IPI00471372.2	WASF1	Wiskott-Aldrich Syndrome Protein Family Member 1	69361	32	293.8	0.05	1.266	1.074
IPI00225670.1	GPHN	Gephyrin	95227	31	260.0	0.07	1.399	1.073
IPI00121833.3	ACAA1A	3-Ketoacyl-Coa Thiolase A, Peroxisomal	49553	13	120.5	0.07	1.819	1.072
IPI00113112.3	RAB3B	Ras-Related Protein Rab-3B	29532	31	152.5	0.71	1.221	1.072
IPI00408909.4	MTAP1A	Isoform 1 of Microtubule-Associated Protein 1A	360950	61	69.1	0.02	0.783	1.067
IPI00830456.1	DPP10	Putative Uncharacterized Protein Dpp10	100667	35	70.7	0.03	0.746	1.066
IPI00224093.2	PHYHIPL	Isoform 1 of Phytanoyl-Coa Hydroxylase-Interacting Protein-Like	51706	11	45.7	0.06	0.566	1.040
IPI00319965.3	PSMD6	26S Proteasome Non-Atpase Regulatory Subunit 6	54006	26	60.0	0.06	0.767	1.034
IPI00122974.1	GPM6A	Neuronal Membrane Glycoprotein M6-A	35824	30	115.2	0.42	1.213	1.030
IPI00111013.1	CTSD	Cathepsin D	54507	44	48.0	0.19	1.252	1.028
IPI00828723.1	ARHGEF7	Isoform G of Rho Guanine Nucleotide Exchange Factor 7	109939	30	230.1	0.06	1.209	1.027
IPI00619995.4	MYO18A	Isoform 3 of Myosin-Xviiia	281702	75	77.9	0.01	0.402	1.025
IPI00153660.4	DLAT	Dihydrolipoyllysine-Residue Acetyltransferase Component of Pyruvate Dehydrogenas	80941	24	94.5	0.08	0.792	1.005
IPI00775867.1	SLC24A2	Solute Carrier Family 24 (Sodium/Potassium/Calcium Exchanger), Member 2 Isoform	89097	15	222.2	0.04	1.227	1.000
IPI00323600.4	CORO1A	Coronin-1A	58638	19	36.3	0.12	1.233	0.993
IPI00330303.7	LOC100046995	Atic Bifunctional Purine Biosynthesis Protein Purh	75338	14	89.4	0.04	1.233	0.989
IPI00850311.1	LOC100044107	Similar To Guanine Nucleotide Binding Protein, Alpha Z Subunit	47366	20	54.1	0.07	1.370	0.987
IPI00311873.5	PPP1CB	Serine/Threonine-Protein Phosphatase Pp1-Beta Catalytic Subunit	44045	17	36.2	0.16	0.795	0.986
IPI00128880.3	IPO4	Importin-4	133096	45	42.8	0.02	1.596	0.984
IPI00131695.3	ALB	Serum Albumin	86519	30	55.5	0.08	1.205	0.981
IPI00464208.3	ATAD3A	Isoform 2 of Atpase Family Aaa Domain-Containing Protein 3	68154	19	58.7	0.05	1.244	0.969
IPI00132966.3	PPID	Peptidyl-Prolyl Cis-Trans Isomerase D	53588	20	79.6	0.06	0.683	0.954
IPI00133387.1	HSBP1	Heat Shock Factor-Binding Protein 1	10430	5	55.5	0.77	0.641	0.944
IPI00319992.1	HSPA5	78 Kda Glucose-Regulated Protein	91352	58	205.4	0.19	0.781	0.940
IPI00118963.1	MRPL12	39S Ribosomal Protein L12, Mitochondrial	27589	7	114.2	0.26	0.669	0.931
IPI00225390.5	COX6B1	Cytochrome C Oxidase Subunit 6B1	13335	9	63.0	0.26	0.703	0.916
IPI00124778.1	BAIAP2	Isoform 2 of Brain-Specific Angiogenesis Inhibitor 1-Associated Protein 2	70869	31	85.6	0.05	0.796	0.915
IPI00658782.4	GUCY1A2	Loc100044212 Guanylate Cyclase 1, Soluble, Alpha 2	96000	35	446.2	0.07	1.437	0.907
IPI00113849.1	CDC42	Isoform 2 of Cell Division Control Protein 42 Homolog	27063	11	49.7	0.26	0.711	0.907
IPI00134746.5	ASS1	Argininosuccinate Synthase	57183	23	59.3	0.12	0.780	0.905
IPI00119138.1	UQCRC2	Cytochrome B-C1 Complex Subunit 2, Mitochondrial	56780	46	142.8	0.12	0.717	0.898
IPI00116112.3	DCTN2	Dynactin Subunit 2	54243	20	37.8	0.19	0.621	0.889
IPI00125501.1	EPB4.1L3	Isoform 1 of Band 4.1-Like Protein 3	124721	46	145.0	0.11	0.791	0.882
IPI00850217.1	LOC100046119	Similar To Ubiquitin-Conjugating Enzyme E2 Ubch-Ben	29691	17	91.2	0.24	0.795	0.880
IPI00379694.4	HMGCL	Hydroxymethylglutaryl-Coa Lyase, Mitochondrial Precursor	41365	12	57.6	0.08	0.659	0.877
IPI00121534.11	CAR2	Carbonic Anhydrase 2	35213	14	123.1	0.43	0.634	0.873
IPI00123058.1	CNTN1	Contactin-1	131512	61	134.7	0.13	1.330	0.870
IPI00221788.3	HPCAL4	Hippocalcin-Like Protein 4	27848	13	90.5	0.40	0.725	0.863
IPI00340165.5	N/A	Putative Uncharacterized Protein Ensmusp00000082571	25217	59	218.7	0.65	0.761	0.859
IPI00378764.6	VGF	Vgf Nerve Growth Factor Inducible	72507	40	264.7	0.05	1.200	0.858
IPI00137730.7	PEBP1	Phosphatidylethanolamine-Binding Protein 1	24943	59	314.5	0.87	0.695	0.855
IPI00356608.4	AAK1	Isoform 2 of Ap2-Associated Protein Kinase 1	109945	24	84.8	0.09	0.702	0.838
IPI00230108.6	PDIA3	Protein Disulfide-Isomerase A3	72918	34	72.7	0.14	1.214	0.807
IPI00929850.1	PGM1	Phosphoglucomutase-1	72920	50	128.7	0.09	0.801	0.799
IPI00128904.1	PCBP1	Poly(Rc)-Binding Protein 1	42550	38	39.0	0.08	0.700	0.799
IPI00136936.1	VPS29	Isoform 1 of Vacuolar Protein Sorting-Associated Protein 29	24913	24	45.1	0.13	0.826	0.797
IPI00420882.3	OGDH	Isoform 4 of 2-Oxoglutarate Dehydrogenase, Mitochondrial	135060	35	70.3	0.05	1.053	0.794
IPI00625955.1	ACSL6	Isoform 1 of Long-Chain-Fatty-Acid--Coa Ligase 6	93291	57	310.4	0.19	0.728	0.792
IPI00315999.4	ATP6V1F	V-Type Proton Atpase Subunit F	15491	31	91.4	0.81	0.939	0.791
IPI00329843.4	ANKFY1	Isoform 1 of Ankyrin Repeat And Fyve Domain-Containing Protein 1	146604	41	145.3	0.02	0.947	0.788
IPI00652758.1	TOM1	Target of Myb Protein 1 Isoform 2	63422	19	94.0	0.11	0.921	0.782
IPI00828891.1	DOCK7	Dedicator of Cytokinesis 7	279411	58	96.8	0.01	1.104	0.781
IPI00224570.3	PRKAR2B	Camp-Dependent Protein Kinase Type Ii-Beta Regulatory Subunit	53534	55	186.7	0.35	0.836	0.781
IPI00850740.1	LOC100044138	Similar To Cdcrel-1Ai Isoform 2	53675	68	164.6	0.35	0.873	0.780
IPI00468481.2	ATP5B	Atp Synthase Subunit Beta, Mitochondrial	63566	175	1390.3	2.35	0.690	0.779
IPI00119346.1	CALB2	Calretinin	39680	25	41.9	0.17	0.872	0.778
IPI00470095.1	GIT1	Arf Gtpase-Activating Protein Git1	98822	39	63.3	0.07	0.674	0.776
IPI00319602.3	CNP	Isoform Cnpii of 2',3'-Cyclic-Nucleotide 3'-Phosphodiesterase	60878	217	1522.5	2.54	0.749	0.766
IPI00465786.3	TLN1	Talin-1	321417	104	38.2	0.01	0.899	0.765
IPI00124639.1	NT5C	5'(3')-Deoxyribonucleotidase, Cytosolic Type	26028	33	271.7	0.13	0.961	0.756
IPI00848608.1	KALRN	Isoform 1 of Kalirin	403375	68	42.7	0.01	0.680	0.740
IPI00377728.2	TOMM70A	Mitochondrial Import Receptor Subunit Tom70	86471	36	520.7	0.08	0.954	0.739
IPI00125266.1	ASAH1	Acid Ceramidase	53254	10	50.4	0.06	0.854	0.739
IPI00125929.2	NDUFA4	Nadh Dehydrogenase [Ubiquinone] 1 Alpha Subcomplex Subunit 4	12059	7	42.9	0.65	0.623	0.736
IPI00227836.1	OLA1	Isoform 2 of Obg-Like Atpase 1	38855	17	41.9	0.09	1.184	0.735
IPI00672824.3	PDP1	[Pyruvate Dehydrogenase [Acetyl-Transferring]]-Phosphatase 1, Mitochondrial	68709	19	77.9	0.10	1.104	0.728
IPI00315593.4	NAGA	Alpha-N-Acetylgalactosaminidase	53497	38	87.2	0.13	0.701	0.728
IPI00321348.3	IGSF8	Immunoglobulin Superfamily Member 8	69856	36	240.3	0.15	0.992	0.726
IPI00122826.1	CEND1	Cell Cycle Exit And Neuronal Differentiation Protein 1	20206	12	84.5	0.36	0.698	0.725
IPI00124225.3	PSME2	Proteasome Activator Complex Subunit 2	34265	20	36.5	0.10	1.023	0.718
IPI00402982.2	TMOD2	Isoform 1 of Tropomodulin-2	50438	15	105.9	0.14	0.842	0.718
IPI00230615.8	NIF3L1	Putative Uncharacterized Protein	47347	14	84.9	0.07	1.242	0.711
IPI00318496.1	GAD1	Glutamate Decarboxylase 1	79819	26	464.4	0.08	0.649	0.710
IPI00154047.1	HIBCH	3-Hydroxyisobutyryl-Coa Hydrolase, Mitochondrial	51509	16	59.2	0.06	0.811	0.695
IPI00421137.3	KIF5C	Kinesin Heavy Chain Isoform 5C	138339	35	59.0	0.05	0.624	0.695
IPI00222008.2	OSCP1	Organic Solute Carrier Protein 1 Isoform	52789	27	198.1	0.06	0.620	0.685
IPI00118986.1	ATP5O	Loc100047429 Atp Synthase Subunit O, Mitochondrial	30098	19	41.1	0.11	1.067	0.680
IPI00461085.3	TMOD2	Isoform 2 of Tropomodulin-2	34267	10	107.8	0.10	0.650	0.675
IPI00875081.1	MTMR1	Myotubularin-Related Protein 1	87567	18	105.0	0.04	0.823	0.674
IPI00229527.7	LTA4H	Leukotriene A-4 Hydrolase	81468	28	246.4	0.04	1.322	0.671
IPI00110426.1	INPP4A	Isoform 1 of Type I Inositol-3,4-Bisphosphate 4-Phosphatase	121500	35	53.3	0.05	0.943	0.671
IPI00316623.4	CTNND1	Isoform 3 of Catenin Delta-1	118891	17	74.9	0.03	1.339	0.664
IPI00153266.1	SCCPDH	Probable Saccharopine Dehydrogenase	56491	6	56.8	0.06	0.791	0.660
IPI00463909.3	HUWE1	E3 Ubiquitin-Protein Ligase Huwe1	545036	83	41.5	0.01	1.258	0.658
IPI00269408.1	SNAP47	Isoform 1 of Synaptosomal-Associated Protein 47	55849	15	43.9	0.06	0.990	0.657
IPI00226521.5	SDR39U1	Isoform 2 of Epimerase Family Protein Sdr39U1	33929	12	54.1	0.10	0.997	0.650
IPI00880426.1	LPHN3	Putative Uncharacterized Protein Lphn3	191340	38	218.8	0.03	0.517	0.646
IPI00757120.2	ANGEL2	Isoform 2 of Protein Angel Homolog 2	69507	17	75.4	0.05	0.995	0.631
IPI00127237.1	PEX14	Peroxisomal Membrane Protein Pex14	46658	8	206.4	0.07	0.854	0.618
IPI00553717.6	MCCC2	Methylcrotonoyl-Coa Carboxylase Beta Chain, Mitochondrial	69820	17	55.9	0.05	0.527	0.602
IPI00626662.3	ALDH1A1	Retinal Dehydrogenase 1	67532	21	60.3	0.15	0.622	0.588
IPI00110265.1	SIRT2	Isoform 1 of Nad-Dependent Deacetylase Sirtuin-2	52069	8	60.4	0.06	0.475	0.519
IPI00263013.4	PLP1	Isoform 1 of Myelin Proteolipid Protein	34810	54	179.7	1.27	0.486	0.509
IPI00330754.1	BDH1	D-Beta-Hydroxybutyrate Dehydrogenase, Mitochondrial	45903	33	213.3	0.07	0.521	0.491
IPI00226563.4	TTYH3	Isoform 1 of Protein Tweety Homolog 3	61384	30	45.0	0.05	0.653	0.487
IPI00651782.2	GBAS	Glioblastoma Amplified	38525	28	114.4	0.18	0.981	0.483
IPI00378681.6	UBR4	Isoform 1 of E3 Ubiquitin-Protein Ligase Ubr4	666494	110	153.6	N/A	0.732	0.393
IPI00762897.2	UGGT1	Udp-Glucose:Glycoprotein Glucosyltransferase 1	207122	36	109.6	0.02	0.650	0.384
