# Supplementary material for: Combining Comparative Proteomics and Molecular Genetics Uncovers Regulators of Synaptic and Axonal Stability and Degeneration In Vivo
Source: PLoS Genet. 2012 Aug 30;8(8):e1002936. doi: 10.1371/journal.pgen.1002936 (PMC3431337; doi:10.1371/journal.pgen.1002936)
Supplement: Table S2 — All proteins with altered expression levels >20% in WldS striatal synaptosome preparations at 24 and/or 48 hrs after cortical lesion. (PDF) [file pgen.1002936.s002.pdf]

*Table S2: All proteins with altered expression levels >20% in WldS striatal synaptosome preparations at 24 and/or 48hrs after cortical lesion*

| Accession Number | | Protein Name | Mol Weight (Da) | Peptides | Score | emPAI | Average Ratio 24Hrs vs Unlesioned | Average Ratio 48Hrs vs Unlesioned |
|---|---|---|---|---|---|---|---|---|
| IPI00223757.4 | AKR1B3 | Aldose Reductase | 44265 | 13 | 34.4 | 0.07 | 1.739 | 2.306 |
| IPI00137087.5 | ARFGEF2 | Adp-Ribosylation Factor Guanine Nucleotide-Exchange Factor 2 | 237721 | 68 | 78.1 | 0.01 | 1.225 | 1.873 |
| IPI00421137.3 | KIF5C | Kinesin Heavy Chain Isoform 5C | 138339 | 35 | 59.0 | 0.05 | 1.483 | 1.801 |
| IPI00330754.1 | BDH1 | D-Beta-Hydroxybutyrate Dehydrogenase, Mitochondrial | 45903 | 33 | 213.3 | 0.07 | 1.503 | 1.739 |
| IPI00221788.3 | HPCAL4 | Hippocalcin-Like Protein 4 | 27848 | 13 | 90.5 | 0.40 | 1.337 | 1.693 |
| IPI00329843.4 | ANKFY1 | Isoform 1 of Ankyrin Repeat And Fyve Domain-Containing Protein 1 | 146604 | 41 | 145.3 | 0.02 | 2.215 | 1.604 |
| IPI00118880.5 | GUCY1B3 | Guanylate Cyclase Soluble Subunit Beta-1 | 81390 | 28 | 70.2 | 0.08 | 1.474 | 1.566 |
| IPI00111218.1 | ALDH2 | Aldehyde Dehydrogenase, Mitochondrial | 66445 | 13 | 60.4 | 0.10 | 1.413 | 1.544 |
| IPI00461857.2 | TBCD | Tubulin-Specific Chaperone D | 151126 | 15 | 40.6 | 0.02 | 1.135 | 1.542 |
| IPI00881197.1 | ME1 | Nadp-Dependent Malic Enzyme | 76100 | 23 | 119.0 | 0.13 | 1.120 | 1.490 |
| IPI00126072.2 | VAT1 | Synaptic Vesicle Membrane Protein Vat-1 Homolog | 49381 | 11 | 109.8 | 0.07 | 1.017 | 1.465 |
| IPI00463909.3 | HUWE1 | E3 Ubiquitin-Protein Ligase Huwe1 | 545036 | 83 | 41.5 | 0.01 | 1.589 | 1.451 |
| IPI00222759.3 | VAT1L | Synaptic Vesicle Membrane Protein Vat-1 Homolog-Like | 55561 | 50 | 191.1 | 0.19 | 1.152 | 1.445 |
| IPI00226790.2 | PIGT | Loc100046871 Gpi Transamidase Component Pig-T | 73344 | 20 | 92.9 | 0.04 | 1.605 | 1.444 |
| IPI00330303.7 | LOC100046995 | Atic Bifunctional Purine Biosynthesis Protein Purh | 75338 | 14 | 89.4 | 0.04 | 1.129 | 1.433 |
| IPI00132966.3 | PPID | Peptidyl-Prolyl Cis-Trans Isomerase D | 53588 | 20 | 79.6 | 0.06 | 1.273 | 1.421 |
| IPI00136936.1 | VPS29 | Isoform 1 of Vacuolar Protein Sorting-Associated Protein 29 | 24913 | 24 | 45.1 | 0.13 | 1.482 | 1.396 |
| IPI00283531.8 | GSTP2 | Glutathione S-Transferase P 2 | 27648 | 25 | 35.0 | 0.41 | 1.093 | 1.358 |
| IPI00461286.1 | PPM1E | Protein Phosphatase 1E | 95287 | 43 | 162.4 | 0.07 | 1.342 | 1.342 |
| IPI00553717.6 | MCCC2 | Methylcrotonoyl-Coa Carboxylase Beta Chain, Mitochondrial | 69820 | 17 | 55.9 | 0.05 | 0.999 | 1.341 |
| IPI00132314.1 | NUCB1 | Nucleobindin-1 | 62502 | 22 | 68.6 | 0.05 | 1.189 | 1.325 |
| IPI00311873.5 | PPP1CB | Serine/Threonine-Protein Phosphatase Pp1-Beta Catalytic Subunit | 44045 | 17 | 36.2 | 0.16 | 1.098 | 1.321 |
| IPI00118131.6 | PPP2R5C | Isoform 2 of Serine/Threonine-Protein Phosphatase 2A 56 Kda Regulatory | 74570 | 32 | 147.2 | 0.04 | 1.379 | 1.310 |
| IPI00626662.3 | ALDH1A1 | Retinal Dehydrogenase 1 | 67532 | 21 | 60.3 | 0.15 | 1.264 | 1.309 |
| IPI00111013.1 | CTSD | Cathepsin D | 54507 | 44 | 48.0 | 0.19 | 1.007 | 1.301 |
| IPI00420426.1 | AP3B2 | Ap-3 Complex Subunit Beta-2 | 143169 | 39 | 82.0 | 0.02 | 1.135 | 1.300 |
| IPI00153740.1 | AHSA1 | Activator of 90 Kda Heat Shock Protein Atpase Homolog 1 | 47143 | 17 | 137.9 | 0.14 | 1.255 | 1.297 |
| IPI00133056.1 | JOSD2 | Josephin-2 | 22208 | 4 | 69.9 | 0.15 | 1.264 | 1.290 |
| IPI00757120.2 | ANGEL2 | Isoform 2 of Protein Angel Homolog 2 | 69507 | 17 | 75.4 | 0.05 | 1.322 | 1.289 |
| IPI00129479.3 | PIP4K2C | Phosphatidylinositol-5-Phosphate 4-Kinase Type-2 Gamma | 57820 | 11 | 55.8 | 0.06 | 1.155 | 1.280 |
| IPI00118821.2 | PAFAH1B2 | Platelet-Activating Factor Acetylhydrolase Ib Subunit Beta | 30299 | 26 | 44.7 | 0.23 | 1.102 | 1.279 |
| IPI00119130.1 | BTBD17 | Btb/Poz Domain-Containing Protein 17 | 55231 | 18 | 295.8 | 0.19 | 1.179 | 1.273 |
| IPI00121534.11 | CAR2 | Carbonic Anhydrase 2 | 35213 | 14 | 123.1 | 0.43 | 1.066 | 1.272 |
| IPI00153903.6 | CLYBL | Citrate Lyase Subunit Beta-Like Protein, Mitochondrial | 46385 | 21 | 66.8 | 0.07 | 0.933 | 1.267 |
| IPI00943452.1 | FLOT2 | Isoform 3 of Flotillin-2 | 52147 | 20 | 34.4 | 0.06 | 1.128 | 1.259 |
| IPI00113772.1 | GABRA1 | Gamma-Aminobutyric Acid Receptor Subunit Alpha-1 | 61683 | 13 | 48.3 | 0.05 | 1.077 | 1.246 |
| IPI00323600.4 | CORO1A | Coronin-1A | 58638 | 19 | 36.3 | 0.12 | 1.225 | 1.240 |
| IPI00875470.1 | PSMD12 | Putative Uncharacterized Protein | 59953 | 40 | 120.7 | 0.05 | 1.057 | 1.239 |
| IPI00850740.1 | LOC100044138 | Similar To Cdcrel-1Ai Isoform 2 | 53675 | 68 | 164.6 | 0.35 | 1.110 | 1.229 |
| IPI00885811.1 | ARL8A | Putative Uncharacterized Protein | 23647 | 19 | 35.3 | 0.14 | 1.054 | 1.224 |
| IPI00132874.4 | MGLL | Putative Uncharacterized Protein | 42726 | 25 | 75.9 | 0.16 | 1.016 | 1.222 |
| IPI00119346.1 | CALB2 | Calretinin | 39680 | 25 | 41.9 | 0.17 | 0.923 | 1.221 |
| IPI00137409.3 | TKT | Transketolase | 81961 | 52 | 127.3 | 0.22 | 1.043 | 1.220 |
| IPI00108125.4 | EIF5A | Eukaryotic Translation Initiation Factor 5A-1 | 21308 | 12 | 137.6 | 0.55 | 1.154 | 1.216 |
| IPI00127237.1 | PEX14 | Peroxisomal Membrane Protein Pex14 | 46658 | 8 | 206.4 | 0.07 | 0.971 | 1.216 |
| IPI00223092.5 | HADHA | Trifunctional Enzyme Subunit Alpha, Mitochondrial | 103683 | 39 | 60.9 | 0.03 | 1.046 | 1.206 |
| IPI00130489.1 | RAB35 | Ras-Related Protein Rab-35 | 29076 | 17 | 174.5 | 0.24 | 1.124 | 1.206 |
| IPI00221577.1 | CPT1C | Carnitine O-Palmitoyltransferase 1, Brain Isoform | 97634 | 19 | 151.1 | 0.03 | 1.064 | 1.203 |
| IPI00110426.1 | INPP4A | Isoform 1 of Type I Inositol-3,4-Bisphosphate 4-Phosphatase | 121500 | 35 | 53.3 | 0.05 | 1.109 | 1.201 |
| IPI00130589.8 | SOD1 | Superoxide Dismutase [Cu-Zn] | 19146 | 30 | 154.4 | 1.25 | 1.282 | 1.197 |
| IPI00229647.5 | TLN2 | Talin-2 | 325317 | 82 | 224.6 | 0.02 | 1.223 | 1.196 |
| IPI00469987.3 | GDA | Guanine Deaminase | 59707 | 39 | 66.9 | 0.17 | 1.356 | 1.184 |
| IPI00118900.1 | PDE1B | Calcium/Calmodulin-Dependent 3',5'-Cyclic Nucleotide Phosphodiesterase | 71130 | 25 | 166.3 | 0.09 | 1.331 | 1.166 |
| IPI00619995.4 | MYO18A | Isoform 3 of Myosin-Xviiia | 281702 | 75 | 77.9 | 0.01 | 1.274 | 1.158 |
| IPI00465786.3 | TLN1 | Talin-1 | 321417 | 104 | 38.2 | 0.01 | 0.876 | 1.156 |
| IPI00118963.1 | MRPL12 | 39S Ribosomal Protein L12, Mitochondrial | 27589 | 7 | 114.2 | 0.26 | 0.835 | 1.151 |
| IPI00187545.4 | PLXNA4 | Plexin-A4 | 250164 | 54 | 85.9 | 0.01 | 1.425 | 1.142 |
| IPI00127942.4 | DSTN | Destrin | 25544 | 23 | 61.9 | 0.28 | 1.288 | 1.134 |
| IPI00127983.1 | TMED2 | Gm10698 Transmembrane Emp24 Domain-Containing Protein 2 | 26151 | 10 | 90.6 | 0.13 | 1.530 | 1.125 |
| IPI00222008.2 | OSCP1 | Organic Solute Carrier Protein 1 Isoform | 52789 | 27 | 198.1 | 0.06 | 1.560 | 1.124 |
| IPI00124639.1 | NT5C | 5'(3')-Deoxyribonucleotidase, Cytosolic Type | 26028 | 33 | 271.7 | 0.13 | 1.539 | 1.110 |
| IPI00918940.1 | STX16 | Syntaxin 16 | 34142 | 17 | 314.9 | 0.10 | 1.297 | 1.108 |
| IPI00136867.1 | SLC6A11 | Sodium- And Chloride-Dependent Gaba Transporter 3 | 79481 | 36 | 197.0 | 0.27 | 0.787 | 1.097 |
| IPI00131695.3 | ALB | Serum Albumin | 86519 | 30 | 55.5 | 0.08 | 1.445 | 1.096 |
| IPI00116112.3 | DCTN2 | Dynactin Subunit 2 | 54243 | 20 | 37.8 | 0.19 | 0.758 | 1.095 |
| IPI00225307.1 | SARS | Seryl-Aminoacyl-Trna Synthetase, Isoform Cra_B | 77460 | 39 | 235.7 | 0.13 | 1.240 | 1.095 |
| IPI00123342.4 | HYOU1 | Hypoxia Up-Regulated Protein 1 | 135372 | 25 | 43.1 | 0.02 | 1.533 | 1.093 |
| IPI00122521.1 | FXR1 | Isoform E of Fragile X Mental Retardation Syndrome-Related Protein 1 | 89598 | 22 | 36.0 | 0.04 | 0.845 | 1.084 |
| IPI00875821.1 | SBF1 | Putative Uncharacterized Protein Sbf1 | 232709 | 33 | 126.4 | 0.04 | 0.824 | 1.061 |
| IPI00408668.2 | DLG1 | Loc100047603 Isoform 1 of Disks Large Homolog 1 | 117797 | 27 | 82.5 | 0.03 | 0.826 | 1.059 |
| IPI00850733.1 | KPNA6 | Kpna6 Protein | 68216 | 15 | 57.5 | 0.05 | 1.312 | 1.059 |
| IPI00115708.1 | PIP4K2A | Phosphatidylinositol-5-Phosphate 4-Kinase Type-2 Alpha | 58215 | 23 | 58.7 | 0.06 | 0.774 | 1.054 |
| IPI00134746.5 | ASS1 | Argininosuccinate Synthase | 57183 | 23 | 59.3 | 0.12 | 0.723 | 1.052 |
| IPI00120030.1 | CRYM | Mu-Crystallin Homolog | 38845 | 19 | 178.2 | 0.28 | 1.233 | 1.051 |
| IPI00138716.1 | RAP2B | Ras-Related Protein Rap-2B | 24370 | 7 | 61.5 | 0.14 | 0.863 | 1.046 |
| IPI00132575.3 | COTL1 | Coactosin-Like Protein | 19698 | 18 | 106.3 | 0.17 | 0.639 | 1.044 |
| IPI00955134.1 | ARPC4 | Putative Uncharacterized Protein | 24068 | 54 | 76.1 | 0.68 | 1.257 | 1.035 |
| IPI00830456.1 | DPP10 | Putative Uncharacterized Protein Dpp10 | 100667 | 35 | 70.7 | 0.03 | 0.849 | 1.031 |
| IPI00318614.9 | IDH2 | Isocitrate Dehydrogenase [Nadp], Mitochondrial | 62890 | 28 | 59.2 | 0.05 | 0.683 | 1.018 |
| IPI00121443.1 | COX6A1 | Cytochrome C Oxidase Polypeptide Via | 13692 | 14 | 71.2 | 0.95 | 0.775 | 1.015 |
| IPI00875081.1 | MTMR1 | Myotubularin-Related Protein 1 | 87567 | 18 | 105.0 | 0.04 | 0.785 | 1.014 |
| IPI00230003.7 | PITPNA | Phosphatidylinositol Transfer Protein Alpha Isoform | 41227 | 27 | 48.5 | 0.17 | 0.839 | 1.013 |
| IPI00124282.1 | RALA | Ras-Related Protein Ral-A | 30402 | 11 | 65.5 | 0.11 | 0.800 | 1.003 |
| IPI00131540.3 | VTI1A | Vesicle Transport Through Interaction With T-Snares Homolog 1A | 28317 | 23 | 115.4 | 0.12 | 0.871 | 0.992 |
| IPI00279443.1 | HSPA12A | Heat Shock 70 Kda Protein 12A | 89768 | 64 | 281.6 | 0.24 | 0.880 | 0.988 |
| IPI00222457.2 | DARS | Aspartyl-Trna Synthetase, Cytoplasmic Isoform 1 | 66998 | 23 | 45.6 | 0.05 | 0.817 | 0.987 |
| IPI00122409.1 | SNAP91 | Isoform Long of Clathrin Coat Assembly Protein Ap180 | 103943 | 47 | 157.6 | 0.17 | 0.872 | 0.987 |
| IPI00762198.2 | HBB-B1 | Beta-Globin | 19931 | 27 | 101.3 | 0.86 | 1.293 | 0.981 |
| IPI00137227.1 | RAB2A | Ras-Related Protein Rab-2A | 27658 | 10 | 98.8 | 0.26 | 0.808 | 0.981 |
| IPI00224570.3 | PRKAR2B | Camp-Dependent Protein Kinase Type Ii-Beta Regulatory Subunit | 53534 | 55 | 186.7 | 0.35 | 1.292 | 0.980 |
| IPI00272033.3 | HIST2H2AC | Histone H2A Type 2-C | 18543 | 9 | 170.1 | 0.95 | 0.731 | 0.979 |
| IPI00133557.1 | TPPP3 | Tubulin Polymerization-Promoting Protein Family Member 3 | 27643 | 7 | 46.0 | 0.12 | 0.855 | 0.970 |
| IPI00651782.2 | GBAS | Glioblastoma Amplified | 38525 | 28 | 114.4 | 0.18 | 0.812 | 0.969 |
| IPI00626790.3 | GLUL | Glutamine Synthetase | 49526 | 62 | 456.6 | 0.57 | 0.766 | 0.967 |
| IPI00652978.4 | 1110020G09RIK | Isoform 4 of Upf0465 Protein C5Orf33 Homolog | 50239 | 32 | 41.7 | 0.07 | 0.877 | 0.963 |
| IPI00227432.7 | DNM3 | Isoform 1 of Dynamin-3 | 115173 | 96 | 201.6 | 0.22 | 0.875 | 0.962 |
| IPI00114241.2 | NEFH | Neurofilament Heavy Polypeptide | 162231 | 15 | 40.7 | 0.04 | 0.720 | 0.960 |
| IPI00125035.1 | AK3L1;AK3L2-PS | Adenylate Kinase Isoenzyme 4, Mitochondrial | 29115 | 10 | 51.7 | 0.11 | 1.252 | 0.956 |
| IPI00153660.4 | DLAT | Dihydrolipoyllysine-Residue Acetyltransferase Component of Pyruvate Deh | 80941 | 24 | 94.5 | 0.08 | 0.849 | 0.951 |
| IPI00109109.1 | SOD2 | Superoxide Dismutase [Mn], Mitochondrial | 29987 | 26 | 223.0 | 0.52 | 0.797 | 0.951 |
| IPI00648312.3 | NT5DC3 | 5'-Nucleotidase Domain-Containing Protein 3 | 74119 | 47 | 304.6 | 0.19 | 0.811 | 0.949 |
| IPI00331579.1 | SYNGR3 | Synaptogyrin-3 | 25686 | 11 | 111.5 | 0.28 | 0.715 | 0.947 |
| IPI00648141.1 | GSK3A | Glycogen Synthase Kinase-3 Alpha | 59328 | 8 | 58.6 | 0.06 | 0.839 | 0.946 |
| IPI00313962.3 | UCHL1 | Ubiquitin Carboxyl-Terminal Hydrolase Isozyme L1 | 30336 | 68 | 1222.4 | 0.87 | 0.834 | 0.943 |
| IPI00125267.4 | VAPA | Vesicle-Associated Membrane Protein-Associated Protein A | 35366 | 13 | 47.4 | 0.09 | 0.822 | 0.942 |
| IPI00881096.1 | NAPG | Putative Uncharacterized Protein Napg | 49418 | 34 | 206.7 | 0.38 | 0.862 | 0.941 |
| IPI00134918.4 | PTPMT1 | Protein-Tyrosine Phosphatase Mitochondrial 1 | 32742 | 9 | 36.8 | 0.10 | 0.827 | 0.933 |
| IPI00319994.6 | LDHA | L-Lactate Dehydrogenase A Chain | 45335 | 104 | 451.4 | 0.76 | 0.875 | 0.932 |
| IPI00468688.4 | TARS | Threonyl-Trna Synthetase, Cytoplasmic | 103684 | 17 | 35.2 | 0.03 | 1.248 | 0.927 |
| IPI00229703.6 | VAMP2 | Vesicle-Associated Membrane Protein 2 | 15173 | 17 | 88.3 | 0.83 | 0.863 | 0.926 |
| IPI00849722.3 | CADM1 | Isoform 5 of Cell Adhesion Molecule 1 | 42702 | 9 | 99.4 | 0.16 | 0.824 | 0.925 |
| IPI00379694.4 | HMGCL | Hydroxymethylglutaryl-Coa Lyase, Mitochondrial Precursor | 41365 | 12 | 57.6 | 0.08 | 1.270 | 0.916 |
| IPI00469392.2 | RTN4 | Isoform 1 of Reticulon-4 | 150472 | 15 | 42.7 | 0.02 | 0.712 | 0.914 |
| IPI00751607.1 | LOC100045958 | Similar To Hcg45299 Isoform 2 | 39500 | 15 | 237.6 | 0.17 | 0.861 | 0.900 |
| IPI00420882.3 | OGDH | Isoform 4 of 2-Oxoglutarate Dehydrogenase, Mitochondrial | 135060 | 35 | 70.3 | 0.05 | 0.837 | 0.900 |
| IPI00762713.1 | DNAJC6 | Isoform 3 of Putative Tyrosine-Protein Phosphatase Auxilin | 123424 | 27 | 41.2 | 0.08 | 0.872 | 0.892 |
| IPI00224740.6 | PFN1 | Profilin-1 | 18161 | 16 | 68.6 | 0.67 | 0.804 | 0.882 |
| IPI00554868.2 | PRPS1 | Ribose-Phosphate Pyrophosphokinase 1 | 41727 | 13 | 73.6 | 0.16 | 0.756 | 0.880 |
| IPI00407130.4 | PKM2 | Isoform M2 of Pyruvate Kinase Isozymes M1/M2 | 69938 | 276 | 2188.0 | 2.96 | 0.863 | 0.876 |
| IPI00130555.1 | RASAL1 | Rasgap-Activating-Like Protein 1 | 101237 | 51 | 372.6 | 0.10 | 0.818 | 0.876 |
| IPI00880839.1 | HSPA9 | Stress-70 Protein, Mitochondrial | 90128 | 66 | 169.0 | 0.20 | 0.869 | 0.874 |
| IPI00119067.3 | TPPP | Tubulin Polymerization-Promoting Protein | 32249 | 46 | 333.2 | 0.98 | 1.282 | 0.873 |
| IPI00115429.1 | GGT7 | Gamma-Glutamyltransferase 7 | 74611 | 23 | 187.8 | 0.04 | 0.761 | 0.867 |
| IPI00116279.3 | CCT5 | T-Complex Protein 1 Subunit Epsilon | 73731 | 32 | 46.2 | 0.04 | 0.823 | 0.862 |
| IPI00318496.1 | GAD1 | Glutamate Decarboxylase 1 | 79819 | 26 | 464.4 | 0.08 | 0.564 | 0.860 |
| IPI00113149.1 | STX1B | Syntaxin-1B | 41665 | 88 | 664.6 | 2.15 | 0.794 | 0.860 |
| IPI00125266.1 | ASAH1 | Acid Ceramidase | 53254 | 10 | 50.4 | 0.06 | 0.835 | 0.857 |
| IPI00467530.3 | ABLIM1 | Isoform 1 of Actin-Binding Lim Protein 1 | 115052 | 29 | 61.7 | 0.03 | 0.794 | 0.853 |
| IPI00111501.1 | CPLX2 | Complexin-2 | 21887 | 44 | 105.2 | 1.35 | 0.783 | 0.838 |
| IPI00116843.1 | MT-ND4 | Nadh-Ubiquinone Oxidoreductase Chain 4 | 56581 | 9 | 52.7 | 0.06 | 1.312 | 0.836 |
| IPI00331541.5 | PFKM | 6-Phosphofructokinase, Muscle Type | 99455 | 52 | 270.0 | 0.14 | 0.779 | 0.836 |
| IPI00122826.1 | CEND1 | Cell Cycle Exit And Neuronal Differentiation Protein 1 | 20206 | 12 | 84.5 | 0.36 | 0.778 | 0.835 |
| IPI00127408.3 | RAC1 | Ras-Related C3 Botulinum Substrate 1, Isoform Cra_A | 30262 | 18 | 35.8 | 0.23 | 0.857 | 0.834 |
| IPI00470981.2 | RTN3 | Isoform 1 of Reticulon-3 | 125432 | 18 | 145.0 | 0.03 | 0.794 | 0.834 |
| IPI00123518.1 | LYPLA2 | Acyl-Protein Thioesterase 2 | 28770 | 8 | 56.7 | 0.12 | 0.714 | 0.826 |
| IPI00408909.4 | MTAP1A | Isoform 1 of Microtubule-Associated Protein 1A | 360950 | 61 | 69.1 | 0.02 | 0.863 | 0.824 |
| IPI00315593.4 | NAGA | Alpha-N-Acetylgalactosaminidase | 53497 | 38 | 87.2 | 0.13 | 0.779 | 0.824 |
| IPI00162781.2 | AHCYL1 | Putative Adenosylhomocysteinase 2 | 71861 | 45 | 137.6 | 0.14 | 1.472 | 0.818 |
| IPI00776358.1 | ACAT3 | Acetyl-Coenzyme A Acetyltransferase 3 | 48288 | 25 | 145.9 | 0.07 | 1.239 | 0.817 |
| IPI00321308.4 | AARS | Alanyl-Trna Synthetase, Cytoplasmic | 127166 | 38 | 178.1 | 0.05 | 0.875 | 0.799 |
| IPI00351246.3 | MPP3 | Maguk P55 Subfamily Member 3 | 75970 | 21 | 179.8 | 0.09 | 1.087 | 0.794 |
| IPI00653237.2 | WDR7 | Wdr7 Protein | 183911 | 70 | 57.6 | 0.05 | 0.978 | 0.790 |
| IPI00405699.2 | ALDH4A1 | Delta-1-Pyrroline-5-Carboxylate Dehydrogenase, Mitochondrial | 72571 | 20 | 133.0 | 0.05 | 0.848 | 0.787 |
| IPI00111556.1 | PPP2CB | Serine/Threonine-Protein Phosphatase 2A Catalytic Subunit Beta Isoform | 39773 | 16 | 36.2 | 0.17 | 0.846 | 0.787 |
| IPI00111151.2 | RPH3A | Rabphilin-3A | 91507 | 31 | 34.7 | 0.11 | 0.953 | 0.784 |
| IPI00828723.1 | ARHGEF7 | Isoform G of Rho Guanine Nucleotide Exchange Factor 7 | 109939 | 30 | 230.1 | 0.06 | 0.948 | 0.783 |
| IPI00132217.1 | FIS1 | Mitochondrial Fission 1 Protein | 21922 | 8 | 92.5 | 0.15 | 0.988 | 0.781 |
| IPI00378764.6 | VGF | Vgf Nerve Growth Factor Inducible | 72507 | 40 | 264.7 | 0.05 | 0.684 | 0.776 |
| IPI00380799.5 | PDE2A | Putative Uncharacterized Protein | 121877 | 39 | 507.4 | 0.05 | 1.062 | 0.775 |
| IPI00454049.4 | ECHS1 | Enoyl-Coa Hydratase, Mitochondrial | 38850 | 20 | 113.7 | 0.09 | 0.891 | 0.774 |
| IPI00135677.2 | EHD3 | Eh Domain-Containing Protein 3 | 74833 | 28 | 128.2 | 0.09 | 1.000 | 0.773 |
| IPI00470095.1 | GIT1 | Arf Gtpase-Activating Protein Git1 | 98822 | 39 | 63.3 | 0.07 | 0.870 | 0.773 |
| IPI00399905.5 | PPFIA3 | Liprin-Alpha-3 | 134308 | 52 | 79.4 | 0.02 | 0.958 | 0.773 |
| IPI00850311.1 | LOC100044107 | Similar To Guanine Nucleotide Binding Protein, Alpha Z Subunit | 47366 | 20 | 54.1 | 0.07 | 0.900 | 0.772 |
| IPI00230615.8 | NIF3L1 | Putative Uncharacterized Protein | 47347 | 14 | 84.9 | 0.07 | 0.610 | 0.771 |
| IPI00380195.1 | PGP | Phosphoglycolate Phosphatase | 39234 | 12 | 53.5 | 0.08 | 1.248 | 0.769 |
| IPI00121440.4 | ETFB | Electron Transfer Flavoprotein Subunit Beta | 36048 | 17 | 78.2 | 0.19 | 0.982 | 0.767 |
| IPI00322312.3 | ARHGDIA | Rho Gdp-Dissociation Inhibitor 1 | 29534 | 26 | 115.5 | 0.38 | 0.767 | 0.765 |
| IPI00153813.4 | EXOC1 | Exocyst Complex Component 1 | 127509 | 42 | 66.4 | 0.05 | 0.977 | 0.765 |
| IPI00848608.1 | KALRN | Isoform 1 of Kalirin | 403375 | 68 | 42.7 | 0.01 | 0.810 | 0.763 |
| IPI00402982.2 | TMOD2 | Isoform 1 of Tropomodulin-2 | 50438 | 15 | 105.9 | 0.14 | 1.118 | 0.757 |
| IPI00471372.2 | WASF1 | Wiskott-Aldrich Syndrome Protein Family Member 1 | 69361 | 32 | 293.8 | 0.05 | 1.249 | 0.757 |
| IPI00310916.1 | CD81 | Cd81 Antigen | 29842 | 8 | 251.9 | 0.37 | 1.070 | 0.755 |
| IPI00122975.1 | GPM6B | Isoform 8 of Neuronal Membrane Glycoprotein M6-B | 36390 | 10 | 93.1 | 0.30 | 1.009 | 0.755 |
| IPI00134353.3 | NOL3 | Nucleolar Protein 3 | 25693 | 6 | 87.4 | 0.13 | 1.125 | 0.755 |
| IPI00129319.3 | PPP1R7 | Protein Phosphatase 1 Regulatory Subunit 7 | 49290 | 28 | 62.9 | 0.14 | 0.831 | 0.755 |
| IPI00404650.2 | ADAM22 | Isoform 3 of Disintegrin And Metalloproteinase Domain-Containing Protein | 114734 | 29 | 47.3 | 0.03 | 0.939 | 0.749 |
| IPI00153266.1 | SCCPDH | Probable Saccharopine Dehydrogenase | 56491 | 6 | 56.8 | 0.06 | 0.804 | 0.746 |
| IPI00273164.1 | ALDH5A1 | Succinate-Semialdehyde Dehydrogenase, Mitochondrial | 66542 | 43 | 126.4 | 0.21 | 0.987 | 0.745 |
| IPI00137313.2 | PLXNA2 | Plexin-A2 | 242332 | 50 | 90.6 | 0.01 | 0.933 | 0.744 |
| IPI00464317.3 | GLS | Glutaminase Isoform 1 | 87852 | 83 | 372.0 | 0.20 | 0.846 | 0.739 |
| IPI00131535.1 | LANCL1 | Lanc-Like Protein 1 | 53050 | 27 | 119.9 | 0.27 | 0.753 | 0.737 |
| IPI00230194.5 | GNG2 | Guanine Nucleotide-Binding Protein G(I)/G(S)/G(O) Subunit Gamma-2 | 10393 | 11 | 131.1 | 0.33 | 0.963 | 0.732 |
| IPI00230680.7 | ATP4A | Potassium-Transporting Atpase Alpha Chain 1 | 130912 | 85 | 309.9 | 0.16 | 0.956 | 0.731 |
| IPI00153176.2 | FBXO2 | F-Box Only Protein 2 | 36678 | 5 | 56.8 | 0.09 | 0.853 | 0.728 |
| IPI00337930.4 | CKAP5 | Isoform 1 of Cytoskeleton-Associated Protein 5 | 292550 | 73 | 257.7 | 0.02 | 0.832 | 0.726 |
| IPI00123465.1 | COPS7A | Isoform 1 of Cop9 Signalosome Complex Subunit 7A | 36157 | 13 | 114.5 | 0.30 | 0.515 | 0.724 |
| IPI00230108.6 | PDIA3 | Protein Disulfide-Isomerase A3 | 72918 | 34 | 72.7 | 0.14 | 1.233 | 0.723 |
| IPI00649135.2 | GSTM1 | Glutathione S-Transferase, Mu 1 | 34793 | 53 | 268.9 | 0.58 | 0.839 | 0.715 |
| IPI00341282.2 | ATP5F1 | Atp Synthase Subunit B, Mitochondrial | 36345 | 24 | 108.2 | 0.09 | 1.127 | 0.714 |
| IPI00118986.1 | ATP5O | Loc100047429 Atp Synthase Subunit O, Mitochondrial | 30098 | 19 | 41.1 | 0.11 | 0.712 | 0.714 |
| IPI00751955.1 | TMX2 | Putative Uncharacterized Protein Tmx2 | 35998 | 17 | 179.4 | 0.09 | 0.880 | 0.714 |
| IPI00331016.1 | SEC24B | Sec24 Related Gene Family, Member B | 148774 | 32 | 38.6 | 0.04 | 0.923 | 0.710 |
| IPI00316623.4 | CTNND1 | Isoform 3 of Catenin Delta-1 | 118891 | 17 | 74.9 | 0.03 | 1.167 | 0.702 |
| IPI00119853.1 | GNAS | Isoform Gnas-1 of Guanine Nucleotide-Binding Protein G(S) Subunit Alpha | 54000 | 19 | 42.8 | 0.13 | 0.892 | 0.695 |
| IPI00377728.2 | TOMM70A | Mitochondrial Import Receptor Subunit Tom70 | 86471 | 36 | 520.7 | 0.08 | 0.712 | 0.694 |
| IPI00263013.4 | PLP1 | Isoform 1 of Myelin Proteolipid Protein | 34810 | 54 | 179.7 | 1.27 | 1.016 | 0.690 |
| IPI00880426.1 | LPHN3 | Putative Uncharacterized Protein Lphn3 | 191340 | 38 | 218.8 | 0.03 | 0.818 | 0.686 |
| IPI00308484.3 | SPARCL1 | Sparc-Like Protein 1 | 86814 | 24 | 40.2 | 0.04 | 1.003 | 0.685 |
| IPI00460132.1 | TUFM | Isoform 2 of Elongation Factor Tu, Mitochondrial | 55368 | 27 | 102.8 | 0.26 | 0.708 | 0.677 |
| IPI00466069.3 | EEF2 | Elongation Factor 2 | 115996 | 28 | 45.2 | 0.06 | 0.757 | 0.675 |
| IPI00762897.2 | UGGT1 | Udp-Glucose:Glycoprotein Glucosyltransferase 1 | 207122 | 36 | 109.6 | 0.02 | 1.106 | 0.666 |
| IPI00675436.2 | GM5422 | Similar To Proteasome (Prosome, Macropain) 26S Subunit, Non-Atpase, 2 | 108022 | 41 | 158.6 | 0.03 | 0.927 | 0.651 |
| IPI00918623.1 | 2310061I04RIK | Novel Protein | 29696 | 13 | 34.0 | 0.11 | 0.726 | 0.650 |
| IPI00221602.1 | SFXN5 | Sideroflexin-5 | 41735 | 19 | 128.7 | 0.26 | 0.939 | 0.644 |
| IPI00556827.1 | ATP2B1 | Plasma Membrane Calcium Atpase 1 | 163010 | 84 | 442.8 | 0.22 | 0.766 | 0.632 |
| IPI00117657.2 | OPA1 | Isoform 1 of Dynamin-Like 120 Kda Protein, Mitochondrial | 137336 | 65 | 366.1 | 0.15 | 0.619 | 0.624 |
| IPI00126048.2 | PSMD13 | Isoform 1 of 26S Proteasome Non-Atpase Regulatory Subunit 13 | 50425 | 14 | 52.6 | 0.07 | 0.750 | 0.623 |
| IPI00461085.3 | TMOD2 | Isoform 2 of Tropomodulin-2 | 34267 | 10 | 107.8 | 0.10 | 1.076 | 0.620 |
| IPI00153400.2 | H2AFJ | Histone H2A.J | 18296 | 12 | 390.5 | 0.66 | 0.529 | 0.597 |
| IPI00133387.1 | HSBP1 | Heat Shock Factor-Binding Protein 1 | 10430 | 5 | 55.5 | 0.77 | 0.978 | 0.596 |
| IPI00459279.2 | QDPR | Dihydropteridine Reductase | 30649 | 21 | 58.0 | 0.51 | 1.220 | 0.584 |
| IPI00464208.3 | ATAD3A | Isoform 2 of Atpase Family Aaa Domain-Containing Protein 3 | 68154 | 19 | 58.7 | 0.05 | 0.684 | 0.565 |
| IPI00652758.1 | TOM1 | Target of Myb Protein 1 Isoform 2 | 63422 | 19 | 94.0 | 0.11 | 0.697 | 0.552 |
| IPI00108783.1 | NF1 | Isoform 2 of Neurofibromin | 375725 | 44 | 158.5 | 0.01 | 0.530 | 0.539 |
| IPI00380956.1 | ABI2 | Abl Interactor 2 | 54585 | 17 | 90.7 | 0.12 | 0.752 | 0.518 |
| IPI00229527.7 | LTA4H | Leukotriene A-4 Hydrolase | 81468 | 28 | 246.4 | 0.04 | 0.954 | 0.449 |
| IPI00918347.1 | PIP5K1C | 76 Kda Protein | 87979 | 30 | 80.1 | 0.08 | 0.625 | 0.441 |
| IPI00265406.3 | PFAS | Phosphoribosylformylglycinamidine Synthase | 159634 | 27 | 137.1 | 0.02 | 0.569 | 0.361 |
| IPI00473728.1 | RPL14 | Putative Uncharacterized Protein | 35236 | 14 | 67.0 | 0.09 | 0.312 | 0.318 |
| IPI00120342.6 | SLC17A6 | Vesicular Glutamate Transporter 2 | 74329 | 23 | 114.8 | 0.04 | 0.605 | 0.316 |
| IPI00626994.3 | IPO5 | Isoform 1 of Importin-5 | 143246 | 37 | 395.2 | 0.05 | 0.293 | 0.298 |
| IPI00828891.1 | DOCK7 | Dedicator of Cytokinesis 7 | 279411 | 58 | 96.8 | 0.01 | 0.638 | 0.256 |
