# Supplementary material for: Combining Comparative Proteomics and Molecular Genetics Uncovers Regulators of Synaptic and Axonal Stability and Degeneration In Vivo
Source: PLoS Genet. 2012 Aug 30;8(8):e1002936. doi: 10.1371/journal.pgen.1002936 (PMC3431337; doi:10.1371/journal.pgen.1002936)
Supplement: Table S3 — Proteins with altered expression levels >20% in striatal synapse-enriched preparations from wild-type mice 48 hrs after cortical lesion (emPAI = exponentially modified protein abundance index). (PDF) [file pgen.1002936.s003.pdf]

Table S3: Proteins with altered expression levels >20% in striatal synapse-enriched preparations from wild-type mice 48hrs after cortical lesion

Symbol	Protein Name	Accession Number	Mol Weight (Da)	emPAI	Peptides Used / Unique	Ratio (Injured:Control)
NFASC	Neurofascin	IPI00329927.4	155723	0.04	3/2	2.03
CSP/DNAJC5	Cystine String Protein/Dnaj Homolog, Subfamily C, Member 5	IPI00875866.1	28769	0.25	4/2	1.76
DCLK1	Doublecortin-Like Kinase 1	IPI00468380.4	100794	0.07	5/2	1.49
DNAJC6	Aunxillin/Dnaj Homolog, Subfamily C, Member 6	IPI00762713.1	123424	0.08	5/3	1.45
ROCK2	Rho-Associated Protein Kinase 2	IPI00108150.1	206952	0.02	1/1	1.45
ABLIM1	Isoform 1 of Actin-Binding Lim Protein 1	IPI00467530.3	115052	0.03	1/1	1.43
ANK3	Ankyrin 3, Node of Ranvier (Ankyrin G)	IPI00623506.3	577303	0.01	4/2	1.43
GLO1	Glyoxalase I	IPI00321734.7	26747	0.42	6/2	1.42
GDA	Guanine Deaminase	IPI00469987.3	59707	0.17	4/3	1.42
CCT7/TCP1	T-Complex Protein 1 Subunit Beta	IPI00331174.5	73208	0.09	1/1	1.40
HIST2H2AC/AB	Histone H2A Type 2-C	IPI00272033.3	18543	0.95	4/2	1.40
HTT	Huntingtin	IPI00271166.4	385122	0.01	1/1	1.39
SGTA	Small Glutamine-Rich Tetratricopeptide Repeat (Tpr)-Containing, Alpha	IPI00116331.1	38788	0.28	7/2	1.38
CFL1	Cofilin 1 (Non-Muscle)	IPI00890117.1	26685	0.80	9/3	1.34
DLG1	Loc100047603 Isoform 1 of Disks Large Homolog 1	IPI00408668.2	117797	0.03	1/1	1.34
WDR7	Wd Repeat Domain 7	IPI00653237.2	183911	0.05	7/3	1.32
GAD2	Glutamate Decarboxylase 2 (Pancreatic Islets And Brain, 65Kda)	IPI00318522.3	77901	0.28	11/4	1.31
D10Jhu81e	Es1 Protein Homolog, Mitochondrial	IPI00133284.1	34499	0.20	3/2	1.31
USP9X	Ubiquitin Specific Peptidase 9, X-Linked	IPI00798468.1	334392	0.02	3/2	1.29
CLTB	Clathrin, Light Chain (Lcb)	IPI00554923.1	28446	0.39	4/3	1.29
RAB2A	Rab2A, Member Ras Oncogene Family	IPI00137227.1	27658	0.26	4/2	1.28
DMXL2	Dmx-Like 2	IPI00896744.1	392746	0.03	11/2	1.26
SPTBN2	Beta Iii Spectrin	IPI00134344.6	309135	0.05	6/5	1.23
MYO5A	Myosin Va (Heavy Chain 12, Myoxin)	IPI00118120.1	267915	0.08	14/6	1.23
NAPG	N-Ethylmaleimide-Sensitive Factor Attachment Protein, Gamma	IPI00881096.1	49418	0.38	12/4	1.22
INA	Internexin Neuronal Intermediate Filament Protein, Alpha	IPI00135965.2	63789	0.42	12/6	1.22
DPYSL4	Dihydropyrimidinase-Like 4	IPI00313151.3	72227	0.14	5/3	1.21
PDXK	Pyridoxal (Pyridoxine, Vitamin B6) Kinase	IPI00283511.1	40753	0.73	17/5	1.20
PGM1/2	Phosphoglucomutase-1	IPI00929850.1	72920	0.09	6/2	0.80
OGDH	Oxoglutarate (Alpha-Ketoglutarate) Dehydrogenase (Lipoamide)	IPI00420882.3	135060	0.05	5/2	0.79
ACSL6	Acyl-Coa Synthetase Long-Chain Family Member 6	IPI00625955.1	93291	0.19	15/3	0.79
ATP6V1F	Atpase, H+ Transporting, Lysosomal 14Kda, V1 Subunit F	IPI00315999.4	15491	0.81	7/2	0.79
PRKAR2B	Protein Kinase, Camp-Dependent, Regulatory, Type Ii, Beta	IPI00224570.3	53534	0.35	19/4	0.78
LOC100044138	Similar To Cdcrel-1Ai	IPI00850740.1	53675	0.35	11/5	0.78
CALB2	Calbindin 2	IPI00119346.1	39680	0.17	3/2	0.78
CNP	2',3'-Cyclic Nucleotide 3' Phosphodiesterase	IPI00319602.3	60878	2.54	87/17	0.77
NDUFA4	Nadh Dehydrogenase (Ubiquinone) 1 Alpha Subcomplex, 4, 9Kda	IPI00125929.2	12059	0.65	3/2	0.74
PDP1	Pyruvate Dehyrogenase Phosphatase Catalytic Subunit 1	IPI00672824.3	68709	0.10	5/2	0.73
NAGA	N-Acetylgalactosaminidase, Alpha-	IPI00315593.4	53497	0.13	7/2	0.73
IGSF8	Immunoglobulin Superfamily, Member 8	IPI00321348.3	69856	0.15	11/3	0.73
GAD1	Glutamate Decarboxylase 1 (Brain, 67Kda)	IPI00318496.1	79819	0.08	5/2	0.71
KIF5C	Kinesin Family Member 5C	IPI00421137.3	138339	0.05	2/2	0.70
HIBCH	3-Hydroxyisobutyryl-Coa Hydrolase, Mitochondrial	IPI00154047.1	51509	0.06	1/1	0.70
INPP4A	Isoform 1 of Type I Inositol-3,4-Bisphosphate 4-Phosphatase	IPI00110426.1	121500	0.05	1/1	0.67
ALDH1A1	Aldehyde Dehydrogenase 1 Family, Member A1	IPI00626662.3	67532	0.15	5/2	0.59
SIRT2	Isoform 1 of Nad-Dependent Deacetylase Sirtuin-2	IPI00110265.1	52069	0.06	1/1	0.52
UBR4	Isoform 1 of E3 Ubiquitin-Protein Ligase Ubr4 (A.K.A P600)	IPI00378681.6	666494	N/A	1/1	0.39
