# Supplementary material for: Combining Comparative Proteomics and Molecular Genetics Uncovers Regulators of Synaptic and Axonal Stability and Degeneration In Vivo
Source: PLoS Genet. 2012 Aug 30;8(8):e1002936. doi: 10.1371/journal.pgen.1002936 (PMC3431337; doi:10.1371/journal.pgen.1002936)
Supplement: Table S4 — Systems level analysis of functional clustering of proteins modified in degenerating synapses (networks identified as statistically significant clusters [P<0.05], IPA analysis). (PDF) [file pgen.1002936.s004.pdf]

*Table S4: Systems level analysis of functional clustering of proteins modified in degenerating synapses (networks identified as statistically significant clusters [P<0.05], IPA analysis)*

| | Functional Annotation | Number of Proteins | % of Candidate List | Proteins |
|---|---|---|---|---|
| **Disease/Disorder** | Neurological Disorder | 35 | 54.7 | ABLIM1, ALDH1A1, ANK3, CALB2, CLTB, CNP, DARS, DCLK1, DNAJC5, DNAJC6, GAD1, GAD2, GLO1, GRM3, HIBCH, HTT, HUWE1, IGSF8, INA, INPP4A, MYO5A, NAGA, NAPG, NRCAM, PDP1, PDXK, PEX14, PIP5K1C, PRKAR2B, PSMD13, ROCK2, SGTA, SPTBN2, UBR4, WDR7 |
| | Metabolic Disorder | 24 | 37.5 | ABLIM1, ALDH1A1, ANK3, ANKFY1, ASAH1, DCLK1, DMXL2, DPYSL5, EPB41L1, GAD2, GRM3, HIBCH, HTT, IVD, MCCC2, NAGA, NIF3L1, NRCAM , OLA1, PDP1, PEX14, PHGDH, TLN1, WDR7 |
| | Movement disorder | 11 | 17.2 | ANK3, CNP, GAD2,MYO5A, PDP1, PDXK, PRKAR2B, ROCK2, SGTA, UBR4, WDR7 |
| | Alzheimer's Disease | 11 | 17.2 | ANK3, CNP, DARS, GAD2, GRM3, HTT, IGSF8, PEX14, PRKAR2B, PSMD13, WDR7 |
| | Neuromuscular Disease | 10 | 15.6 | CNP, GAD2, MYO5A, PDP1, PDXK, PRKAR2B, ROCK2, SGTA, UBR4, WDR7 |
| | Huntingtons's Disease | 5 | 7.8 | GAD2, MYO5A, PDP1, ROCK2, SGTA |
| | Parkinson's Disease | 5 | 7.8 | CNP, PDXK, PRKAR2B, UBR4, WDR7 |
| | Schizophrenia | 5 | 7.8 | CLTB, CNP, GAD1, GAD2, PRKAR2B |
| **Cellular Process** | Synaptic Transmission | 7 | 10.9 | CNP, DNAJC5, GAD1, GAD2, GRM3, HTT, MYO5A |
| | Neurogenesis | 7 | 10.9 | ACSL6, DCLK1, DPYSL4, DPYSL5, GDA, HTT, PLXNA4 |
| | Modification of Amino Acids | 7 | 10.9 | CFL1, DCLK1, GAD1, GAD2, PDP1, PRKAR2B, ROCK2 |
| | Guidance of Axons | 6 | 9.4 | ABLIM1, ANK3, DPYSL5, KIF5C, NFASC, NRCAM |
| | Formation of Filaments | 6 | 9.4 | CFL1, CNP, HTT, INA, ROCK2, TLN1 |
| | Development of Neurites | 5 | 7.8 | CNP, DCLK1, PHGDH, PIP5K1C, ROCK2 |
| | Assembly of Filaments | 5 | 7.8 | CFL1, CNP, HTT, ROCK2, TLN1 |
| | Biogenesis of Cytoskeleton | 5 | 7.8 | CFL1, CNP, INA, ROCK2, TLN1 |
| | Inhibition of Apoptosis | 5 | 7.8 | CFL1, GLO1, HTT, NOL3, PDXK |
| | Exocytosis | 4 | 6.3 | DNAJC5, HTT, MYO5A, PIP5K1C |
| | Transport of Vesicles | 4 | 6.3 | HTT, MYO5A, NAPG, SPTBN2 |
| | Movement of Neurons | 4 | 6.3 | CFL1, DCLK1, GAD1, PIP5K1C |
| | Biogenesis of Synapse | 3 | 4.7 | ANK3, MYO5A, NFASC |
| | Quantity of Phosphatidylinositol Diphosphate | 3 | 4.7 | INPP4A, PIP5K1C, ROCK2 |
| | Formation of Vesicles | 3 | 4.7 | HTT, PIP5K1C, ROCK2 |
| | Import of Protein | 3 | 4.7 | CFL1, HTT, PEX14 |
| | Biogenesis of Actin Cytoskeleton | 3 | 4.7 | CFL1, ROCK2, TLN1 |
| | Proliferation of Neurons | 3 | 4.7 | ACSL6, CFL1, CNP |
| | Neurodegeneration | 3 | 4.7 | CNP, DNAJC5, HTT |
| | Development of Axons | 3 | 4.7 | CNP, DCLK1, PIP5K1C |
